# A biophysical model linking cortical scalar potentials and polarization waves to slow traveling activity in vision

**DOI:** 10.1038/s41598-026-56500-x

**Published:** 2026-06-09

**Authors:** Hyun Myung Jang, Youngwoo Jang, Hyeon Han

**Affiliations:** 1 Research Institute of Advanced Materials, Seoul National University, Seoul, 08826 Republic of Korea; 2 Pohang University of Science and Technology (POSTECH), Pohang, 37673 Republic of Korea; 3 Department of Cardiology, Gil Medical Center, Gachon University College of Medicine, Incheon, 21565 Republic of Korea; 4 Department of Materials Science and Engineering, Pohang University of Science and Technology (POSTECH), Pohang, 37673 Republic of Korea; 5 Korea-Max Planck-PSI Center for Quantum Emergent Spintronics (KOMQUEST), Pohang, 37673 Republic of Korea; 6 Max Planck Institute of Microstructure Physics, Weinberg 2, 06120 Halle (Saale), Germany

**Keywords:** Polarization waves, Scalar potential field, Cortical modulation wave, Primary visual cortex, Linear convolution structure, Visual cognition, Biological techniques, Biophysics, Neuroscience, Physics

## Abstract

Recent experimental studies indicate that visual cognition is accompanied by slowly propagating biophysical traveling waves in cortical tissue. Here, we propose that polarization waves within the visual cortex provide the biophysical basis for the observed slowly propagating traveling waves. For this, we first compute the propagation speed of scalar potential fields generated by impressed ionic currents in primary visual cortex using a theoretically derived telegraph-type model equation. On the basis of the linear convolution framework, we then show that the scalar potential field $\boldsymbol{\phi\:}(\boldsymbol{x},\boldsymbol{t})$ and the polarization wave $\boldsymbol{P}(\boldsymbol{x},\boldsymbol{t})$, arising from slowly oscillating neuronal dipoles, propagate with the same velocity. Remarkably, the predicted speed is consistent with the independently estimated propagation speed of an effective cortical modulation wave (~ 1.5 cm/s). Since each parallel retinal/LGN/geniculocortical pathway gathers signals from more than a hundred photoreceptors, the resulting response is likely to cover a range of effective cortical wave numbers. In this multi-$\boldsymbol{k}$ regime, we show that dispersive spreading naturally emerges over time, which may reduce cross-channel interference and help stabilize perceptual processing.

**Supplementary Information:**

The online version contains supplementary material available at 10.1038/s41598-026-56500-x.

## Introduction

**Fig. 1:**
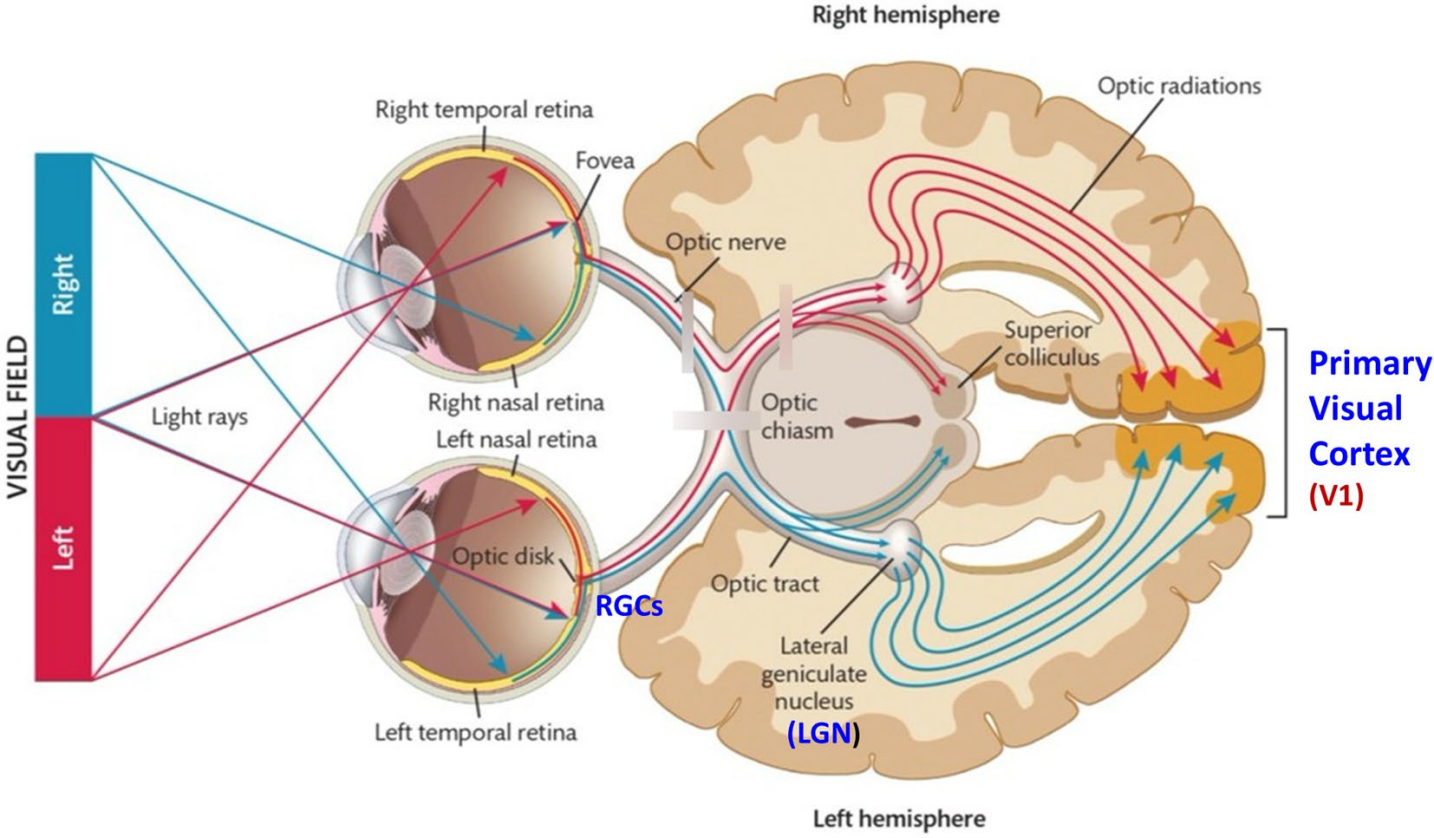
Standard anatomical visual pathway and model entry point in V1. Visual information is first converted into electrochemical neural signals in the retina through phototransduction and retinal processing. These include (i) photon absorption by retinal photopigments *via* $\pi\:-{\pi\:}^{*}$ electronic transitions, (ii) opsin conformational change, (iii) activation of the G-protein cascade, (iv) modulation of cGMP-gated channels, (v) photoreceptor hyperpolarization, (vi) altered glutamate release, and (vii) On/OFF bipolar cells activation for RGC (retinal ganglion cell) spike control. Integrated RGC signals are transmitted through optic nerve channels. RGC axons partially decussate at the optic chiasm and continue to project through the optic tracts to the lateral geniculate nucleus (LGN). Geniculocortical projections then reach the primary visual cortex (V1) *via* the optic radiations. The present model begins at this cortical stage: the input considered here is not the incident optical electromagnetic wave, but the post-retinal, geniculocortically delivered effective cortical input to V1. This effective input is modeled as generating impressed ionic currents, scalar-potential fields, and polarization responses in the cortical medium. The anatomical pathway schematic was adapted from Miquel Perello Nieto, *Human visual pathway.svg* (Wikimedia Commons, 2015), licensed under CC BY-SA 4.0. Modifications include added labels, and explanatory annotations.

Approximately 90% of the information humans acquire from the external world is visual in origin. Nonetheless, the biophysical mechanism underlying the slowly propagating traveling waves observed in cortical tissue has yet to be fully elucidated. In the present work, we propose a biophysical model for visual cognition in terms of distributed synaptic/impressed currents and associated polarization responses in the primary visual cortex (V1) region. Here, we do **not** assume that optical photons, optical frequencies, or optical wave numbers are transmitted through the optic nerve or directly processed by V1. This is because photons are absorbed in retinal photoreceptors, where opsin-mediated phototransduction converts optical input into changes in membrane potential and synaptic release. The signals reaching V1 are therefore electrochemical neural signals, not optical waves. Our model begins at this cortical stage and treats the resulting synaptic/impressed ionic currents as sources of extracellular scalar potentials and associated polarization-field responses. More detailed descriptions of the standard anatomical visual pathway and our model entry point in V1 are given in Fig. 1 and its caption.

Our study begins by estimating an effective modulation frequency inferred from visual persistence $\left({\omega\:}_{vp}\right)$ that characterizes the effective temporal scale available to neural processing. Thus, we define ${\omega\:}_{vp}=2\pi\:/{t}_{ps}\approx\:150{\hspace{0.17em}}{\mathrm{s}}^{-1}$ as the effective cortical modulation frequency, where ${t}_{ps}$ denotes the temporal persistence of visual perception. This quantity should **not** be interpreted as the perceived frequency of the incident light, but rather as a phenomenological low-frequency neural timescale relevant to post-retinal cortical processing. In contrast, the oscillation frequency of incoming optical waves is extremely high: for green light, a typical angular frequency is $\omega\:\approx\:$ 3.5 × 10¹⁵ s⁻¹, corresponding to a photon frequency $f=\omega\:/2\pi\:\:$≈ 5.6 × 10¹⁴ Hz. Green LED spectra exhibit a half-width at half-maximum (HWHM) that is approximately 3% of their central optical frequency^1–3^. Treating the HWHM as an effective uncertainty ${\Delta\:}\omega\:$ in the photon frequency, we obtain ${\Delta\:}\omega\:\approx\:0.03\omega\:$ ≈ 1.1 × 10¹⁴ s⁻¹ (${\Delta\:}f$ ≈ 1.7 × 10¹³ Hz). On the other hand, the temporal persistence of a green visual image in humans is ~ 40 ms (typically 30–50 ms)^4^. This determines a cortical modulation frequency ${f}_{\mathrm{v}\mathrm{p}}=1/{t}_{ps}\approx\:\frac{1}{0.04{\hspace{0.17em}}\mathrm{s}}\approx\:25{\hspace{0.17em}}\mathrm{H}\mathrm{z},\:{\:\omega\:}_{\mathrm{v}\mathrm{p}}=2\pi\:{f}_{\mathrm{v}\mathrm{p}}\approx\:150{\hspace{0.17em}}{\mathrm{s}}^{-1}.$ Therefore, the effective cortical modulation frequency inferred from visual persistence $\left({\:\omega\:}_{\mathrm{v}\mathrm{p}}\right)$ is exceedingly small compared with both the optical frequency and even the uncertainty $\left({\Delta\:}\omega\:\right)$ in the optical frequency: ${\omega\:}_{\mathrm{v}\mathrm{p}}\approx\:150\ll\:{\Delta\:}\omega\:\approx\:1.1\times\:{10}^{14}<\omega\:\approx\:3.5\times\:{10}^{15} \; {s}^{-1}\mathrm{.}$ Therefore, visible light itself cannot be treated as a “physically resolved entity” by the nervous system. Instead, visible light interacts with the rapidly fluctuating dipole moments of electronic clouds—whose natural oscillations lie in the same $\sim{10}^{15}\:{s}^{-1}$ range—thereby inducing electronic transitions^5–7^.

Because biological organisms perceive the world through the constraints of their own sensory and neural architectures, a view emphasized in organism-centered and brain-centered accounts of perception^8–10^, visual experience should be understood here as a post-retinal neural construction rather than as direct access to the physical optical carrier itself. In the present work, this point is used only to motivate a phenomenological cortical timescale: the effective cortical modulation frequency inferred from visual persistence, ${\omega\:}_{vp}\approx\:150{\hspace{0.17em}}{\mathrm{s}}^{-1}$, is not the perceived frequency of incident light, but a low-frequency scale relevant to post-retinal cortical processing. This effective modulation scale is comparable to the timescale of slow traveling activity reported in visual and cortical systems^11–14^. However, the precise biophysical nature of such slowly propagating cortical traveling waves remains unresolved.

Here, we propose a theoretical framework in which the effective cortical modulation wave results in the propagating polarization wave packet in the primary visual cortex (V1) region of the visual information processing path (Fig. 1). The following sections outline our research and discoveries. **(i)** We first introduce a heuristic estimate of an effective low-frequency cortical modulation scale relevant to post-retinal visual processing. More specifically, we show that the propagation velocity of this modulated wave is ~ 1.5 cm/s, which is similar to or slightly lower than the experimentally observed values ($1.6\sim2.2\:cm/s$) of traveling waves in the visual cortex^15,16^. **(ii)** We extract the propagation velocity of the scalar potential field generated by impressed ionic currents in V1 by numerically simulating the space-time map based on a telegraph-type differential equation derived in this work. **(iii)** Exploiting the linear convolution framework^17–19^, we show that this scalar field $\phi\:(x,t)$ and the polarization field $P\left(x,t\right),$ arising from slowly oscillating neuronal dipoles, propagate with the same velocity. Notably, this velocity agrees well with the independently predicted velocity of the effective cortical modulation wave (~ 1.5 cm/s). **(iv)** Because the ionic influx within a single visual pathway reflects the integrated activity of more than a hundred photoreceptors^20,21^, the resulting polarization field necessarily spans a range of wave numbers. We analyze such a multi-*k* polarization wave packet and show that its amplitude exhibits dispersive spreading in time. This spreading can play a functional role: by temporally separating signals arriving from adjacent visual pathways, it may help suppress cross-channel interference and thereby stabilize visual perception. For this mechanism to be effective, the decaying lifetime ${\tau\:}_{lf}$ must be comparable to or shorter than the inter-channel time delay.

## Results

### Heuristic estimate of an effective cortical modulation scale

**Fig. 2:**
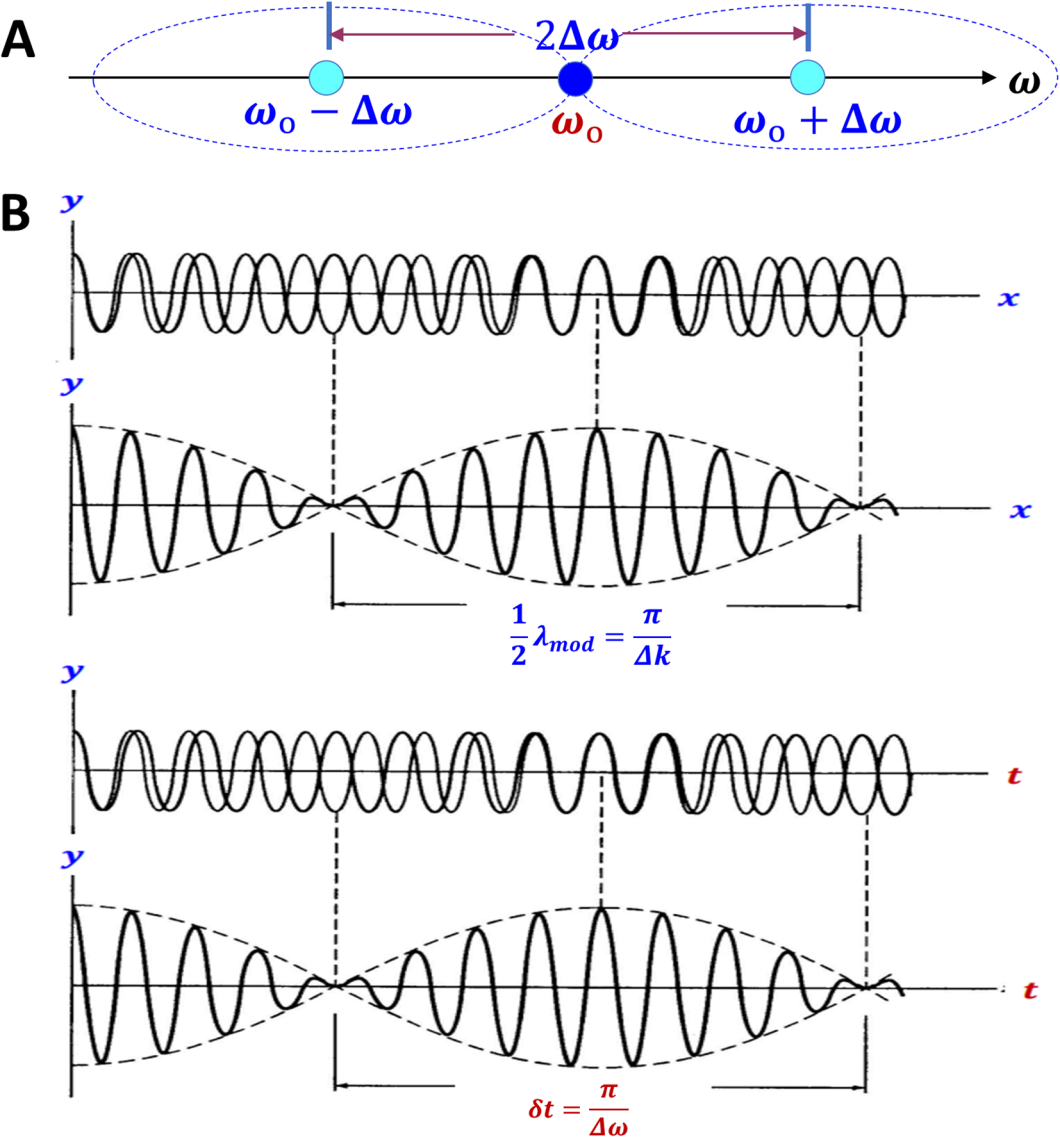
Formation of a modulated wave by superposition of two waves. (**A**) Schematic representation of two simultaneously incident photon waves of identical amplitude but slightly different frequencies, ${\omega\:}_{o}-{\Delta\:}\omega\:$ and ${\omega\:}_{o}+{\Delta\:}\omega\:.$ Under this condition, ${\Delta\:}\omega\:$ can be viewed as the maximal perceptual frequency difference and, thus, as the uncertainty limit in the angular frequency $\omega\:.$ (**B**) (Upper Panel) Position (*x*)-dependent representation of a modulated wave or beat pattern formed by the superposition of two waves of nearly equal wavelengths. As indicated in the figure, half-wavelength of the modulated envelope ($\:\frac{1}{2}{\lambda\:}_{mod}\:$) is equal to $\frac{\pi\:}{{\Delta\:}k}\:,$ where ${\Delta\:}k$ denotes the wave number of the modulated wave, ${k}_{mod}.$ (Lower Panel) Time (*t*)-dependent representation of a modulated wave or beat pattern formed by the superposition of two waves of nearly equal angular frequencies, ${\omega\:}_{o}-{\Delta\:}\omega\:$ and ${\omega\:}_{o}+{\Delta\:}\omega\:$. As indicated in the figure, the time (${\updelta\:}t$) required for the oscillation of $\pi\:$ radian in the beat pattern is equal to $\frac{\pi\:}{{\Delta\:}\omega\:}$. This is because $\delta\:t/{\Delta\:}t\approx\:\delta\:t\:{\Delta\:}\omega\:=\pi\:$ according to Heisenberg’s uncertainty relation. As explained in the text, ${\Delta\:}\omega\:$ can be interpreted as the effective cortical modulation frequency in vision. Similarly, ${\Delta\:}k$ can be viewed as the effective cortical spatial wave-number scale. These quantities refer to post-retinal cortical modulation, not to optical-frequency propagation.

The following derivation of a cognitively relevant modulated wave is **not** intended to describe optical-wave propagation in the visual pathway. This modulated wave (${y}_{\mathrm{m}\mathrm{o}\mathrm{d}}$) is introduced as a heuristic estimate of an effective low-frequency cortical modulation scale after retinal phototransduction and geniculocortical processing. The relevant wave equation can be obtained by considering two components of an effective cortical modulation of identical amplitude A but slightly different frequencies, ${\omega\:}_{o}-{\Delta\:}\omega\:$ and ${\omega\:}_{o}+{\Delta\:}\omega\:$ (Fig. 2A). Their superposition yields a modulated wave with an envelope oscillating at ${\Delta\:}\omega\:.$ Here, ${\Delta\:}\omega\:$ can be defined as effective neural/cortical frequency scale, not optical frequency discrimination. Importantly, the wave number *k* used below is not the optical wave number of the incident light. It denotes an effective spatial Fourier component of the cortical field or population-level response along the cortical surface. The following derivation should therefore be read as a heuristic representation of an effective cortical modulation after retinal phototransduction and geniculocortical transmission, **not** as a description of optical photon propagation through the visual pathway.

Let the displacements of the two sinusoidal waves be ${y}_{1}\left(x,t\right)$ and ${y}_{2}\left(x,t\right).$ Their sum becomes $y\left(x,t\right)={y}_{1}\left(x,t\right)+{y}_{2}\left(x,t\right)=A\left(cos\alpha\:+cos\beta\:\right)$, where $\alpha\:$ and $\beta\:$ are defined as $\alpha\:=\left({\omega\:}_{0}+{\Delta\:}\omega\:\right)t-\left({k}_{0}+{\Delta\:}k\right)x,\:\:\beta\:=({\omega\:}_{0}-{\Delta\:}\omega\:)t-({k}_{0}-{\Delta\:}k)x.$ Using the identity $\mathrm{cos}\alpha\:+\mathrm{cos}\beta\:=2\mathrm{cos}\left[\frac{\alpha\:+\beta\:}{2}\right] \cdot \:\mathrm{cos}\left[\frac{\alpha\:-\beta\:}{2}\right],$ we obtain

1 $$\begin{array}{cccc}&\:y\left(x,t\right)=2A\:\mathrm{c}\mathrm{o}\mathrm{s}({\omega\:}_{0}t-{k}_{0}x){\hspace{0.17em}}\mathrm{c}\mathrm{o}\mathrm{s}({\Delta\:}\omega\:t-{\Delta\:}kx),&\:&\:\end{array}$$

as illustrated in Fig. 2B. Here the first cosine represents the carrier wave, whereas the second cosine defines the slowly varying modulation envelope.

2 $$\:\begin{array}{cccc}&\:{y}_{\mathrm{m}\mathrm{o}\mathrm{d}}(x,t)=2A\mathrm{c}\mathrm{o}\mathrm{s}({\Delta\:}\omega\:t-{\Delta\:}kx).&\:&\:\end{array}$$

The modulation envelope in Eq. (2) is characterized by an effective angular frequency ${\Delta\:}{\omega\:}_{\mathrm{e}\mathrm{f}\mathrm{f}}$. Since the temporal persistence of visual perception, ${t}_{ps}$, provides a finite temporal window for post-retinal cortical processing, we define the effective cortical modulation frequency as

3 $${\Delta\:}{\omega\:}_{\mathrm{e}\mathrm{f}\mathrm{f}}\equiv\:{\omega\:}_{vp}=\frac{2\pi\:}{{t}_{ps}}\approx\:150{\hspace{0.17em}}{\mathrm{s}}^{-1},$$

where ${\Delta\:}{\omega\:}_{\mathrm{e}\mathrm{f}\mathrm{f}}$ is equal to ${\Delta\:}\omega\:$ in Eq. (2). Thus, the uncertainty, ${\Delta\:}\omega\:,$ in Eq. (2) can be effectively read as an effective modulation frequency for describing post-retinal cortical modulation (${\omega\:}_{vp}$). Equivalently, the corresponding Fourier time scale is $\delta\:{t}_{F}\equiv\:\frac{1}{{\Delta\:}{\omega\:}_{\mathrm{e}\mathrm{f}\mathrm{f}}}=\frac{{t}_{ps}}{2\pi\:},$ so that ${\Delta\:}{\omega\:}_{\mathrm{e}\mathrm{f}\mathrm{f}}\:\delta\:{t}_{F}\approx\:1$. This relation should be understood as a phenomenological time–frequency scale inferred from visual persistence, not as a claim that V1 resolves an optical frequency difference. In other words, ${\omega\:}_{vp}\:(\equiv\:{\Delta\:}{\omega\:}_{\mathrm{e}\mathrm{f}\mathrm{f}})$ is not an optical frequency difference resolved by V1, but a phenomenological low-frequency scale used to describe post-retinal cortical modulation.

On the other hand, the corresponding spatial modulation scale can be determined based on the smallest detectable spatial separation, ${\varDelta\:x}_{min}\approx\:{10}^{-4}m,$ using the uncertainty-based relation ($\varDelta\:p\varDelta\:x=\hslash\:{\Delta\:}k{\Delta\:}x\:\sim\:\hslash\:$):

4 $$\varDelta\:k=\frac{1}{{\Delta\:}x}\approx\:\frac{1}{{\varDelta\:x}_{min}}={10}^{+4}\:{m}^{-1}$$

The wave number ${\Delta\:}k$ introduced here is not the optical wave number of incident light. It denotes an effective cortical spatial Fourier scale $k\:[\equiv\:\varDelta\:k\:$in Eq. (2) associated with the post-retinal population-level response along the cortical surface. The estimate ${\Delta\:}k\sim\:1/{\Delta\:}{x}_{\mathrm{m}\mathrm{i}\mathrm{n}}$ should therefore be understood as an order-of-magnitude spatial uncertainty estimate, not as an optical propagation relation. Thus, the velocity of the effective cortical modulation envelope associated with the post-retinal response is obtained from Eq. (2), namely, ${v}_{m}=\frac{{\Delta\:}\omega\:}{{\Delta\:}k}\approx\:\frac{150}{{10}^{4}}=1.5\times\:{10}^{-2}\:\left(m/s\right)=1.5\:\left(cm/s\right).$ This characteristic velocity is $\sim{10}^{10}$ times slower than the speed of light; indeed, $\frac{{v}_{m}}{c}=\frac{1.5}{3\times\:{10}^{10}}\approx\:5\times\:{10}^{-11}.$ If one attempted to interpret this as a slowed electromagnetic wave, the corresponding refractive index of the optic-radiation medium would need to be on the order of $n\sim\:2\times\:{10}^{10}$—an impossibility, since biological tissue in this region has a refractive index of only ≈ 1.4 (Ref.^22^). Therefore, the modulated wave relevant to visual cognition ($\varDelta\:\omega\:$ ≈ 150 s⁻¹, ${\Delta\:}k\:$≈ 10⁴ m⁻¹, ${v}_{m}\:$≈ 1.5 cm/s) cannot be a transverse electromagnetic wave; it must be a fundamentally different, bioelectrically mediated phenomenon.

On the basis of the above examinations, we propose the following view: The modulated input wave that underlies visual cognition is not the external optical wave itself but an internally generated representation. To put it another way, we suggest that the nervous system does not directly track optical carrier oscillations. This modulated wave may arise either (i) as an effective cortical representation generated after retinal and geniculocortical processing, or (ii) as a bioelectric scalar wave emerging from the synaptic and transmembrane ionic currents associated with geniculocortical and intracortical processing. Its propagation velocity (~ 1.5 cm/s) and quasi-static nature indicate that it is fundamentally distinct from the transverse electromagnetic waves of incoming light. Thus, the relevant wave in the present model is not an optical wave but an effective cortical modulation wave generated after phototransduction and geniculocortical transformation. We again emphasize that Eq. (2) is used for heuristic estimates only, not for photon-wave propagation after phototransduction. We will show later that ${y}_{\mathrm{m}\mathrm{o}\mathrm{d}}(x,t)$ effectively plays a role of the bioelectric input wave in the V1 cell layer for visual cognition.

### Cortical traveling waves and scalar potential fields.

**Fig. 3:**
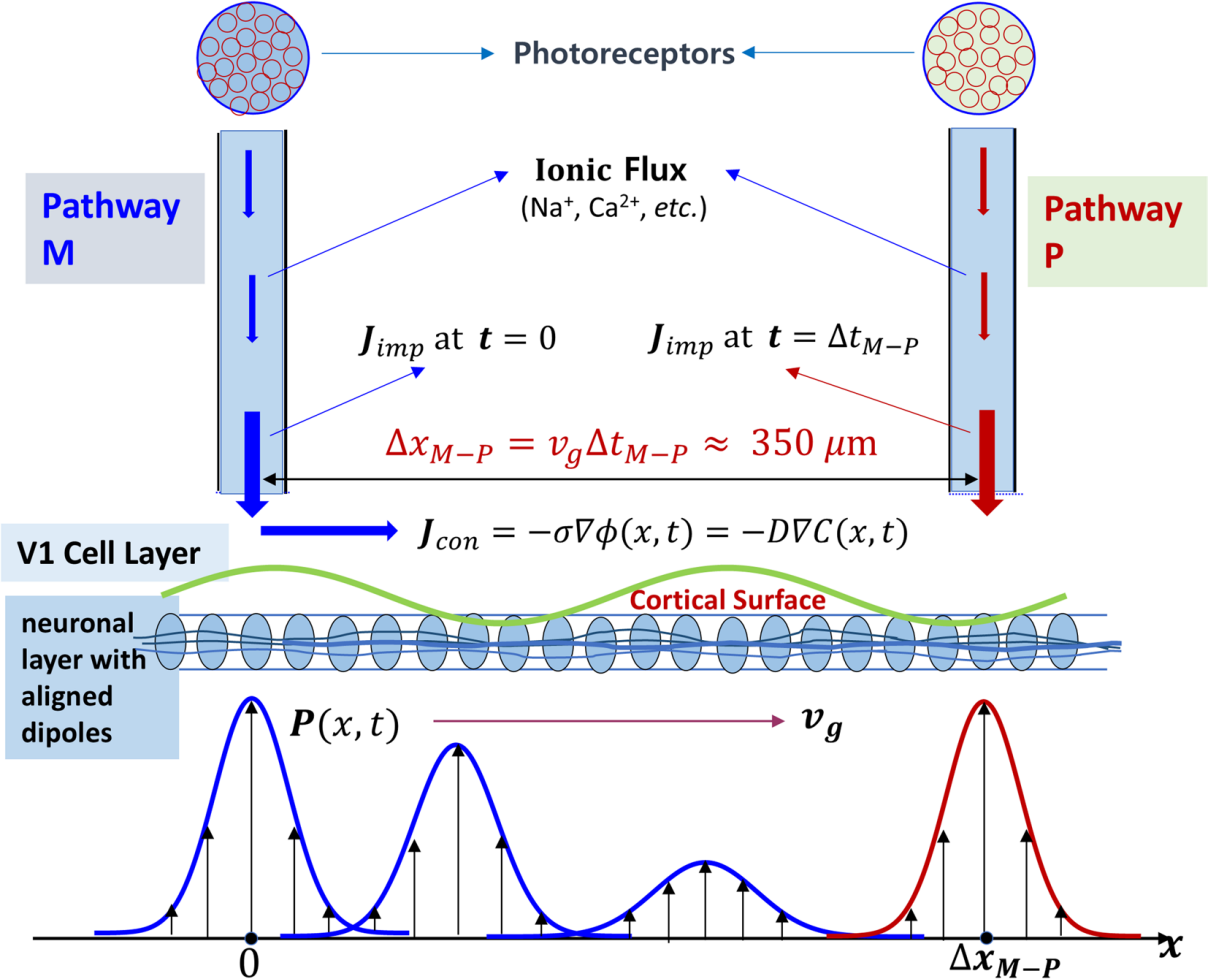
Schematic representation of the primary visual cortex (V1) region. Visual information carried by RGCs reaches the V1 region *via* multiple visual pathways arranged in parallel. Only two representative visual pathways, M and P, are displayed in this schematic figure. Since ${\boldsymbol{J}}_{con}=-\sigma\:\nabla\:\phi\:(x,t)$, the conduction current ${\boldsymbol{J}}_{con}$ along +*x*-direction is proportional to $\nabla\:P(x,t)$ according to the linear convolution theorem. The scalar potential $\phi\:(x,t)$ is measured on the one-dimensional strip along *+x*-axis located at a constant vertical distance ${r}_{o}$ above the neuronal layer in which travelling dipoles are aligned perpendicular to *x*-axis. As marked with blue curves in the bottom panel, the polarization wave packet *P(x,t)* formed at *x=0*, i.e., at the exit of the M channel (at time *t=0*) moves along *+x*-axis but gradually spreads with the passage of time due to non-zero $\beta\:$ (Eq. 13). At $t={\varDelta\:t}_{M-P}$, a new polarization wave packet is formed at the exit of P channel which is $\varDelta\:{x}_{M-P}$ away from the exit of the M channel. This new wave packet centered at $\varDelta\:{x}_{M-P}$ is marked with red color. Therefore, we establish the following relation between $\varDelta\:{x}_{M-P}$ and the inter-channel time delay: $\varDelta\:{x}_{M-P}={v}_{g}{\varDelta\:t}_{M-P}.$

Visual information encoded by retinal ganglion cell (RGC) activity is relayed to the LGN, and V1 subsequently receives geniculocortical input through the optic radiation (Fig. 1). The final signals arriving in the V1 cell layer take the form of ionic fluxes (Fig. 3). Representative parallel visual pathways include the magnocellular (M) and parvocellular (P) pathways. Because each channel has its own axonal conduction velocity and synaptic delay, the arrival times of visual signals differ measurably across channels. These converging signals in V1 have been reported to constitute the primary source for traveling waves propagating along the cortical surface^11–14^. Sato et al.^11^, through a systematic review of previous works, argued that the existence of traveling waves that can influence spike responses in V1 is well established. More recently, Benigno et al.^12^ directly observed traveling waves traversing the entire visual cortex, while Davis et al.^13^ showed that horizontal cortical connections strongly shape intrinsic traveling waves that regulate sensory-evoked responses and perceptual sensitivity. While research confirms that traveling waves exist and play a role in V1, their exact biophysical properties are still unclear. In the present work, we propose—*via* a step-by-step theoretical analysis—that these traveling waves may be described, at coarse-grained bioelectric level, as slowly propagating polarization waves *P(x,t)* moving along the cortical surface (Fig. 3). We will further show that the cortical scalar potential field $\phi\:(x,t)$, which is linearly related to *P(x,t)* through linear convolution, propagates across V1 with the same velocity as *P(x,t)*.

We now consider the physicochemical behavior of the ionic flux (Na^+^, Ca^2+^) injected into the V1 region (Fig. 3). This ion flux generates a large impressed current ${\boldsymbol{J}}_{\mathrm{i}\mathrm{m}\mathrm{p}}$, and the high ion concentration near the input region at *x=0* gives rise to a chemical-potential gradient $\nabla\:\mu\:$ along the *x*-axis near *x=0*. Writing the local chemical potential as $\mu\:\left(x\right)\approx\:{\mu\:}_{0}+RT\mathrm{ln}C\left(x\right)$, we obtain

5 $$\begin{array}{cccc}&\:\nabla\:\mu\:\left(x\right)=\frac{RT}{C\left(x\right)} {\hspace{0.17em}}\nabla\:C\left(x\right)=-{\hspace{0.17em}}\frac{RT}{C\left(x\right)} {\hspace{0.17em}}\frac{\boldsymbol{J}}{D},&\:&\:\end{array}$$

where *D* is the diffusion coefficient of the ions, *R* is the gas constant (8.314 J·K^− 1^·mol^− 1^), and *T* is the absolute temperature. The last equality in Eq. (5) follows from Fick’s first law, $\boldsymbol{J}=-D\nabla\:C$. Because $\nabla\:\mu\:\left(x\right)$ carries a negative sign, Eq. (5) implies that the ionic current tends to flow in the *+x* direction (Fig. 3). The corresponding scalar-potential gradient $\nabla\:\phi\:\left(x\right)$ induced by this current can be written as $\boldsymbol{J}=\sigma\:\boldsymbol{E}=-\sigma\:\nabla\:\phi\:\left(x\right),$ where $\sigma\:$ is the effective conductivity. Substituting this expression into Eq. (5) yields $\nabla\:\mu\:\left(x\right)=\frac{\sigma\:}{D}{\hspace{0.17em}}\frac{RT}{C\left(x\right)}{\hspace{0.17em}}\nabla\:\phi\:\left(x\right).\:$ This relation shows that the chemical-potential gradient and the scalar-potential gradient are directly interrelated with each other. Consequently, when ions diffuse from regions of high chemical potential to regions of low chemical potential along the *+x* direction for $x\ge\:0$ (Fig. 3), the front of the high-$\phi\:\left(x\right)$ region also advances along the *+x*-axis. In short, the $\phi\:(x,t)$ field creates a front that travels mostly parallel to the cortical surface.

Having obtained a qualitative picture of how $\phi\:(x,t)$ moves, we next consider the inter-channel time delay ${\Delta\:}t$ (Ref. ^23–25^) which is crucially important in stabilizing visual perception. Schmolesky et al.^23^ report that the time delay between signals in an M pathway and those in an adjacent P pathway is approximately 23 ms (= 63 − 40 ms) while Maunsell et al.^24^ report a broader range of 10–30 ms. We will show that the time delay ${\Delta\:}t$ should be larger than the effective lifetime (${t}_{lf}$) of the polarization wave packet in V1. If we adopt the reported value by Schmolesky et al.^23^, we can write the following requirement: ${\Delta\:}t=23{\hspace{0.17em}}\:ms\:>\:{t}_{lf}$. If this inequality were violated, the residual visual information from the M pathway would mix with newly arriving information from the P pathway, leading to cross-channel interference in visual perception. The Discussion section provides more in-depth explanations for this inequality relationship in visual perception.

### Dynamics of the scalar potential field and its propagation velocity

In this section, we analyze the propagation velocity of the scalar potential field $\phi\:(x,t)$ in the cortical medium by performing simulations based on the telegraph-type differential equation. We then compare this velocity with the characteristic propagation speed of the effective cortical modulation wave, ${v}_{m}=\frac{{\Delta\:}\omega\:}{{\Delta\:}k}=1.5{\hspace{0.17em}}\mathrm{c}\mathrm{m}/\mathrm{s}$. If the two velocities match, this would imply that the scalar potential field in V1 shares the same effective cortical modulation scale, not optical frequency/wave number, as the modulated wave ${y}_{\mathrm{m}\mathrm{o}\mathrm{d}}(x,t)$ relevant to visual cognition. Since $\phi\:(x,t)$ is linearly linked to the cortical polarization field through a convolution framework, both the potential field $\phi\:(x,t)$ and the polarization wave are expected to move at an identical velocity (${v}_{p}$). Therefore, if two velocities (${v}_{m}$ and ${v}_{p}$) match, this suggests that there exists an input-response relation between ${y}_{\mathrm{m}\mathrm{o}\mathrm{d}}(x,t)$ and the polarization wave *P(x,t)* *via* a linear convolution structure with ${y}_{\mathrm{m}\mathrm{o}\mathrm{d}}(x,t)$ being the input and $P\left(x,t\right)$ as the response. More specifically writing, $P\left(x,t\right)=\int\:\chi\:\left(x-{x}^{{\prime\:}}\right){y}_{\mathrm{m}\mathrm{o}\mathrm{d}}\left({x}^{{\prime\:}},t\right)\:dx{\prime\:}$, where the kernel $\chi\:\left(x-{x}^{{\prime\:}}\right)$ represents dipolar susceptibility in *x*-space. Thus, if the cortical traveling waves in V1 exhibit a speed of approximately $v\approx\:1.5{\hspace{0.17em}}\mathrm{c}\mathrm{m}/\mathrm{s}$, which is nearly identical to ${v}_{m},$ this supports the hypothesis that the experimentally observed cortical traveling waves^15,16^ may correspond to polarization-field responses, and, thus, the observed cortical traveling waves are responding to the input modulated wave which is relevant to visual cognition in V1.

To derive the governing equation for the scalar potential ridge, we combine the condition of charge continuity with the relaxation dynamics of dipole alignment. In an isotropic, homogeneous cortical medium operating in the low-frequency regime, where the high-frequency component of the displacement current can be neglected, the divergence of the total current vanishes, and the total current consists of four contributions: (i) conduction current, (ii) displacement current, (iii) polarization current, and (iv) impressed ionic current generated by neural activity. Thus, we write

6 $$\begin{array}{cccc}&\:\nabla\:\cdot\:{\boldsymbol{J}}_{\mathrm{tot}}=0,\:\:{\:\:\:\:\:\:\boldsymbol{J}}_{\mathrm{tot}}=\sigma\:\boldsymbol{E}+\epsilon\:\frac{\partial\:\boldsymbol{E}}{\partial\:t}+\frac{\partial\:\boldsymbol{P}}{\partial\:t}+{\boldsymbol{J}}_{imp}&\:&\:\end{array}$$

Here, $\sigma\:$ is the tissue conductivity, $\epsilon\:$is the dielectric permittivity, $\boldsymbol{P}$ is the polarization density, and ${\boldsymbol{J}}_{imp}$ is the impressed ionic current. The electric field is defined as $\boldsymbol{E}=-\nabla\:\phi\:$. Since the dipolar polarization in cortical tissue responds at low frequency, we adopt a Debye-type relaxation description:

7 $$\begin{array}{cccc}&\:{\tau\:}_{p}\frac{\partial\:\boldsymbol{P}}{\partial\:t}+\boldsymbol{P}\:={\epsilon\:}_{0}\chi\:\mathbf{E},&\:&\:\end{array}$$

where $\chi\:$ is the effective dielectric susceptibility, ${\epsilon\:}_{0}=8.854\times\:{10}^{-12}\:{J}^{-1}{C}^{2}{m}^{-1}$ the permittivity of free space, and ${\tau\:}_{p}$ the dipole-alignment relaxation time. Assuming a single $\left(k,\omega\:\right)$-mode for $\boldsymbol{P}(x,t)$ and neglecting phase-lag effects in the low-frequency limit (${e}^{i\delta\:}=1$), we can eliminate $\boldsymbol{P}$ from Eqs. (6) and (7). Following a series of algebraic manipulations (see Supplementary Information for details), we arrive at a scalar-potential equation commonly referred to as the telegraph-type partial differential equation (PDE), or the Goldstein–Kac telegraph Equations^26,27^:

8 $$\:\begin{array}{cccc}&\:\tau\:\frac{{\partial\:}^{2}\phi\:}{\partial\:{t}^{2}}+\frac{\partial\:\phi\:}{\partial\:t}=D{\nabla\:}^{2}\phi\:+S(x,t),&\:&\:\end{array}$$

where $\tau\:\equiv\:{\tau\:}_{\mathrm{e}\mathrm{f}\mathrm{f}}\left(k\right)={\tau\:}_{p}\left(\frac{{P}_{0}}{{\phi\:}_{0}}\right){\left(2{\epsilon\:}_{0}\chi\:k\right)}^{-1}$ denotes the effective modal relaxation time, $D=\frac{\sigma\:}{{C}_{\mathrm{e}\mathrm{f}\mathrm{f}}}=\sigma\:{\left(2{\epsilon\:}_{0}\chi\:\:{k}^{2}\right)}^{-1}$ is the effective diffusion coefficient, ${C}_{\mathrm{e}\mathrm{f}\mathrm{f}}$ is an effective capacitance factor, and $S\left(x,t\right)=\frac{1}{{C}_{\mathrm{e}\mathrm{f}\mathrm{f}}}\left|\nabla\:\cdot\:{\boldsymbol{J}}_{imp}\left(x,t\right)\right|$ is the source term associated with the divergence of the impressed ionic current, ${\boldsymbol{J}}_{imp}$. It should be emphasized that ${\tau\:}_{p}$ is the microscopic dipole-alignment relaxation time, whereas $\tau\:$, i.e., ${\tau\:}_{\mathrm{e}\mathrm{f}\mathrm{f}}\left(k\right)$ in Eq. (8) is an effective modal relaxation time obtained after reducing the coupled polarization–scalar-potential dynamics to a single spatial Fourier component. The *k*-dependence of ${\tau\:}_{\mathrm{e}\mathrm{f}\mathrm{f}}$ therefore reflects the mode-dependent field-response geometry of the coarse-grained equation and should not be interpreted as a wave-number-dependent intrinsic material relaxation time.

Under the single-mode *ansatz*, $\phi\:(x,t)={\phi\:}_{0}{e}^{-i(kx-\omega\:t)}$. Substituting this form into Eq. (8) yields the dispersion condition: $-\tau\:{\omega\:}^{2}+i\omega\:+D{k}^{2}=0.$ In the regime $4\tau\:D{k}^{2}\gg\:1$, the solution becomes

9 $$\begin{array}{cccc}&\:\omega\:=\pm\:\sqrt{\frac{D}{\tau\:}}{\hspace{0.17em}}k+\frac{i}{2\tau\:}.&\:&\:\end{array}$$

Thus, the propagation speed of the scalar potential field is $v=\frac{\omega\:}{k}=\sqrt{D/\tau\:}.$ Taking the temporal persistence inferred from the visual lifetime as the effective relaxation time ($\tau\:=42$ ms) and using the modulated-wave velocity ${v}_{m}=1.5\:$ cm/s, we obtain an effective diffusion constant $D={v}^{2}\tau\:\approx\:9.45\times\:{10}^{-6}{\hspace{0.25em}\hspace{0.05em}}{\mathrm{m}}^{2}/\mathrm{s},$ consistent with experimentally measured cortical conductivity–permittivity values^28^. For multi-$\left(k,\omega\:\right)$-mode wave packets, the simulated propagation velocity may differ from $1.5{\hspace{0.17em}}\mathrm{c}\mathrm{m}/\mathrm{s}$. Because the damping term ${e}^{-t/\left(2\tau\:\right)}$ ($={e}^{+i\omega\:t}$) suppresses high-*k* modes (since $\tau\:\propto\:1/k$), the center of the surviving wave-packet spectrum can shift slightly.

**Fig. 4:**
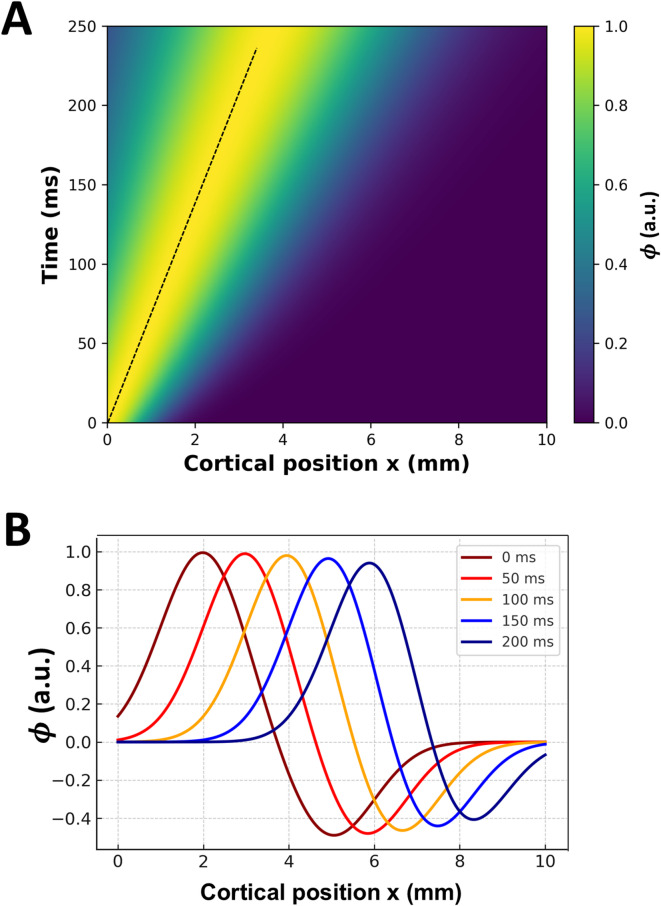
Cortical scalar potentials simulated using the telegraph-type equation. (**A**) Space-time map of the cortical scalar potential field $\phi\:(x,t)$ simulated using the telegraph-type partial differential equation, Eq. (8). Here, $\phi\:\left(x,t\right)$ denotes the scalar-potential field, and $v\:\:$is the ridge velocity extracted from the slope of the space–time trajectory. The nearly linear ridge trajectory is marked with a black broken line. The propagation velocity of the scalar potential ridge estimated using this trajectory line is $v=1.5{\hspace{0.17em}}\:cm/s.$ It is interesting to note that this simulated ridge velocity nearly coincides with the characteristic velocity of the effective cortical modulation wave, ${v}_{m}=1.5\:cm/s.$ (**B**) Snapshot profiles of $\phi\:(x,t)$ at various times, showing the temporal drift of the peak. The propagation velocity estimated using the peak values of $\phi\:(x,t)$ is approximately equal to the propagation velocity of $\phi\:(x,t)$ field obtained from the trajectory line in the above space-time map.

Figure 4A shows the solution of Eq. (8) in 1-D when two visual-pathway inputs (i.e., two geniculocortical input streams), separated by 0.4 mm, deliver synaptic ion influxes with an arbitrary temporal delay of 30 ms. The front velocity was estimated by linear regression of the $\phi\:\left(x,t\right)$ ridge in the space–time map. The simulation is grid-convergent: halving ${\Delta\:}x,\:\:{\Delta\:}t$ changed *v* by < 1%. The space–time map exhibits diagonal ridges, whose slope yields a propagation velocity *v=\:*1.5 cm/s. The nearly linear ridge trajectory indicates that the propagation velocity remains stable over time, confirming a constant-velocity traveling ridge. The central conclusion from this figure is that the scalar-potential ridge propagates at $v=1.5{\hspace{0.17em}}\:\mathrm{c}\mathrm{m}/\mathrm{s}$. Thus, the simulated ridge velocity is equal to the characteristic velocity of the effective cortical modulation wave ${v}_{m}=\frac{{\Delta\:}\omega\:}{{\Delta\:}k}$. Thus, the cortical scalar-potential field shares the same fundamental $\left(\omega\:,\:k\right)$-structure as the effective cortical modulation wave. By the linear convolution theorem, the polarization-wave ridge is expected to propagate at the same speed under the linear, translationally invariant approximation. Consequently, experimentally observed cortical traveling waves with comparable velocities may be interpreted, within the present coarse-grained model, as possible polarization-field responses. These polarization-field responses are responding to the input modulated wave, in accordance with the input-response relation *via* a linear convolution. Figure 4B displays snapshot profiles of $\phi\:(x,t)$ showing the temporal drift of the peak, where $\phi\:(x,t)$ denotes the scalar-potential field. From the ridge displacement between *t=0* and *t=50* ms, the simulated ridge (peak-drift) velocity is estimated as: $v\approx\:\frac{0.8{\hspace{0.17em}}\mathrm{m}\mathrm{m}}{50{\hspace{0.17em}}\mathrm{m}\mathrm{s}}=1.6{\hspace{0.17em}}\mathrm{c}\mathrm{m}/\mathrm{s}.$ This estimate aligns closely with: (i) the trajectory-derived value from the space–time map, and (ii) the uncertainty-based prediction ${v}_{m}=$ 1.5 cm/s. Thus, both methods confirm a robust propagation velocity of *v≈* 1.5–1.6 cm/s.

### Linear convolution relation between scalar potential and polarization

Let us consider a one-dimensional strip along the *x*-axis at a fixed observation depth ${z}_{0}$, located at a constant vertical distance ${r}_{0}$ above the neuronal layer in which dipoles are aligned (Fig. 3). The scalar potential measured on this strip, $\phi\:(x,t)$, is related to the polarization wave *P(x,t)*, which travels along the *x*-direction with the propagation velocity *v*, through a linear convolution. This convolution relation can be written as

10 $$\begin{array}{cccc}&\:\phi\:(x,t;{z}_{0})=\int\:K(x-{x}^{{\prime\:}};{z}_{0}){\hspace{0.17em}}P({x}^{{\prime\:}},t){\hspace{0.17em}}d{x}^{{\prime\:}}=\left(K*P\right)(x-vt)&\:&\:\end{array}$$

Equation (10) expresses velocity preservation: although the kernel *K* may smooth the waveform, it does not alter the phase or propagation speed of the traveling polarization wave. Thus, $\phi\:(x,t)$ inherits the same front velocity as *P(x,t)*. In other words, $P\left(x,t\right)$ and $\phi\:(x,t)$ are kinematically linked, forming an object-shadow relation. Therefore, they propagate with the same velocity. Applying forward Fourier transform to Eq. (10) yields the linear convolution relation in *k*-space:

11 $$\begin{array}{cccc}&\:\phi\:(k,t)=K\left(k\right)P(k,t).&\:&\:\end{array}$$

Here $$P\left(k,t\right)=\int\:P\left({x}^{{\prime\:}},t\right){e}^{+ik{x}^{{\prime\:}}}d{x}^{{\prime\:}},\:\:K\left(k\right)=\int\:K(x-{x}^{{\prime\:}}){e}^{+ik(x-{x}^{{\prime\:}})}dx.$$.

If the polarization wave consists of a single *k*-mode, its Fourier transform is $P\left(k,t\right)=\int\:{P}_{0}{e}^{-i\left({k}_{0}{x}^{{\prime\:}}-\omega\:t\right)}{e}^{+ik{x}^{{\prime\:}}}d{x}^{{\prime\:}}=2\pi\:{P}_{0}{e}^{+i\omega\:t}\delta\:\left({k}_{o}-k\right).$ Substituting this relation into Eq. (11) gives $\phi\:(k,t)$, and inverse Fourier transformation yields $\phi\:\left(x,t\right)=\int\:\phi\:(k,t){e}^{-ikx}dk={\phi\:}_{0}{e}^{-i\left({k}_{0}x-\omega\:t\right)}$, where${\:\:\phi\:}_{0}=2\pi\:{P}_{0}\:K\left({k}_{0}\right).$ Thus, the amplitude of the scalar potential ${(\phi\:}_{0})$ depends solely on the value of the kernel at the carrier wave number, $K\left({k}_{0}\right).$ Consequently, the peak propagation speed of $\phi\:(x,t)$ is identical to that of the polarization wave and is given by $v=\omega\:/{k}_{o}.$ For a translationally invariant single-mode wave, application of the convolution theorem yields

12 $$\left(K*{e}^{-i{k}_{0}x}\right)\left(x\right)={e}^{-i{k}_{0}x}\int\:K(x-x{\prime\:}){e}^{+i{k}_{0}(x-{x}^{{\prime\:}})}dx=K\left({k}_{0}\right){e}^{-i{k}_{0}x}$$

$K\left({k}_{0}\right)$ was obtained by using the definition of $K\left(k\right)$ given in Eq. (11). The right-hand side is equal to $\left(2\pi\:{P}_{0}{e}^{+i\omega\:t}{)}^{-1}\phi\:\right(x,t)$, demonstrating that any translationally invariant kernel with a nonzero Fourier component at the relevant wave number is permissible for $\phi\:(x,t)$ to be a traveling wave as long as its Fourier transform satisfies $K\left({k}_{0}\right)\ne\:0$. In our case, we have already shown that $K\left({k}_{0}\right)={\phi\:}_{0}/\left(2\pi\:{P}_{0}\right)$.

As noted earlier, each anatomical visual pathway receives ion-influx signals originating from more than 100 photoreceptors, representing diverse wavelengths and temporal patterns^20,21^. Thus, the V1 travelling wave is expected to reflect multiple ($k,\omega\:$) components, not a single mode. In such a case, the polarization wave packet may be written as $P(k,t)=P\left(k\right){e}^{+i\omega\:\left(k\right)t}={P}_{0}{\hspace{0.17em}}{e}^{-\alpha\:(k-{k}_{0}{)}^{2}}{e}^{+i\omega\:\left(k\right)t},$ where $\alpha\:=1/\left(2{\sigma\:}^{2}\right)$ and $\sigma\:$ is the standard deviation of the *k*-distribution. The scalar potential satisfies an analogous form: $\phi\:(k,t)={\phi\:}_{0}{e}^{-\alpha\:(k-{k}_{0}{)}^{2}}{e}^{+i\omega\:\left(k\right)t}.$ Substituting these expressions into Eq. (11) shows that $K\left(k\right)={\phi\:}_{0}/{P}_{0}\ne\:0$, which is not restricted to $k={k}_{o}$. Therefore, the scalar potential field $\phi\:(x,t)$ is also a wave packet moving with the same velocity as *P(x,t)* over the spectral range in which $K\left(k\right)$ is nonzero and sufficiently smooth.

To determine the propagation speed of the wave-packet peak, consider the Taylor expansion of $\omega\:\left(k\right)$ about ${k}_{0}$ (Ref.^29^):

$$\omega\:\left(k\right)=\omega\:\left({k}_{0}\right)+(k-{k}_{0}){\left(\frac{d\omega\:}{dk}\right)}_{{k}_{0}}+\frac{1}{2}(k-{k}_{0}{)}^{2}{\left(\frac{{d}^{2}\omega\:}{d{k}^{2}}\right)}_{{k}_{0}}$$

13 $$=\:{\omega\:}_{0}+(k-{k}_{0}){v}_{g}+\frac{1}{2}(k-{k}_{0}{)}^{2}\beta\:,$$

where ${v}_{g}=(d\omega\:/dk{)}_{{k}_{0}}$ is the group velocity, $\beta\:={\left(\frac{{d}^{2}\omega\:}{d{k}^{2}}\right)}_{{k}_{0}}$ is the dispersion coefficient. Intuitively, this wave packet can be regarded as a localized cortical response formed by the superposition of many nearby spatial Fourier components around a central wave number ${k}_{o}$. If all these components propagated with exactly the same velocity, the packet would move without changing its shape. In general, however, the angular frequency $\omega\:\left(k\right)$ depends on *k*, so nearby components travel with slightly different phase velocities. The first derivative ${v}_{g}=(d\omega\:/dk{)}_{{k}_{0}}$ determines the velocity of the packet envelope, whereas the second derivative $\beta\:={\left(\frac{{d}^{2}\omega\:}{d{k}^{2}}\right)}_{{k}_{0}}$ measures how strongly neighboring *k*-components separate from one another during propagation. Thus, ${v}_{g}$ controls the drift of the wave-packet center, while $\beta\:$ controls dispersive spreading and the associated decrease of peak amplitude. Using Eq. (13) in the expression for *P(k,t)* and performing the inverse Fourier transform of *P(k,t)* to real space yields$P(x,t)=P\left({k}_{0}\right){e}^{-i({k}_{0}x-{\omega\:}_{0}t)}A\left(t\right){\hspace{0.17em}}\mathrm{e}\mathrm{x}\mathrm{p}\left[-\frac{{\left(x-{v}_{g}t\right)}^{2}}{4\left(\alpha\:-\frac{i\beta\:t}{2}\right)}\right],$ where $A\left(t\right)$ is a complex prefactor (Supplementary Information for derivation). An identical result is obtained for $\phi\:(x,t)$. The exponential envelope is maximized when $x={v}_{g}t,$ showing that both the polarization and scalar-potential wave packets propagate with the same group velocity ${v}_{g}$. This also supports our estimate of $v\approx\:1.6\:{\mathrm{cm/s}}$ from the peak-drift velocity of $\phi\:(x,t)$ profile presented in Fig. 4B.

### Thermodynamic driving force of slowly moving polarization waves

Up to this point, we have not explicitly identified the thermodynamic mechanism that drives the slow tangential motion of the polarization wave *P(x,t)*. We have argued qualitatively that ionic diffusion from regions of high to low chemical potential would produce a scalar-potential front propagating along the + x-axis. We now build an explanation for how this gradient also drives the polarization wave. From Fick’s law and Ohm’s law, concentration and potential gradients satisfy $D\nabla\:C(x,t)={\upsigma\:}\nabla\:\phi\:(x,t).$ We previously showed that *P(x,t)* is mediated by $\phi\:(x,t)$ according to the relation that $\nabla\:P\left(x,t\right)=\frac{1}{2\pi\:\:K\left({k}_{0}\right)}\nabla\:\phi\:\left(x,t\right).$ Substituting this into the first expression gives

14 $$\begin{array}{cccc}&\:\nabla\:P(x,t)=\frac{1}{2\pi\:K\left({k}_{0}\right)}\left(\frac{D}{\sigma\:}\right)\nabla\:C(x,t)=\frac{1}{2\pi\:K\left({k}_{0}\right)}\left(\frac{b}{\sigma\:}\right)C(x,t)\nabla\:\mu\:(x,t),&\:&\:\end{array}$$

where $\nabla\:\mu\:=(RT/C)\nabla\:C$ and *b* denotes the ionic mobility. The first form of Eq. (14) shows that polarization gradients are directly proportional to concentration gradients. Thus, a spatially varying ionic concentration $\nabla\:C(x,t)$ produces a polarization gradient, which in turn generates a conduction current ${\boldsymbol{J}}_{con}\propto\:-\nabla\:P.$ We can explain more quantitatively by rewriting the first expression of Eq. (14) in the following form: $\nabla\:P\left(x,t\right)=\frac{1}{2\pi\:K\left({k}_{0}\right)}\left(-\frac{1}{\sigma\:}\right){\boldsymbol{J}}_{con}.$ We used Fick’s first law to obtain this expression. According to this relation, the tangential conduction current, ${\boldsymbol{J}}_{\mathrm{c}\mathrm{o}\mathrm{n}}\propto\:-\sigma\:\nabla\:P\left(x,t\right),$ is maximal near the propagating front. This region of maximal current moves together with the polarization-wave front at the same propagation speed (Fig. 4B). When the dominant $({k}_{0},{\omega\:}_{0})$-mode is considered, the resulting polarization-wave front moves with the velocity $v=\frac{{\omega\:}_{0}}{{k}_{0}}$. As ions diffuse from the channel exit at *x=0*, $\nabla\:C(x,t)$ gradually decreases, causing the polarization gradient—and hence the driving force of the traveling wave—to diminish over time (Fig. 3).

The second form of Eq. (14) comes from expressing the tangential conduction current using chemical potential^30,31^: ${\boldsymbol{J}}_{\mathrm{c}\mathrm{o}\mathrm{n}}=-D\nabla\:C\left(x,t\right)=-bC\left(x,t\right)\nabla\:\mu\:\left(x,t\right),$ with *b* denoting ionic mobility. By eliminating $\nabla\:C$ and applying $\frac{{P}_{0}}{{\phi\:}_{0}}=\frac{1}{2\pi\:K\left({k}_{0}\right)}$, we obtain Eq. (14). Because $\nabla\:\mu\:$ has the dimensions of force, chemical-potential gradients represent the ultimate thermodynamic driving force of polarization-wave propagation. We now find the mobility *b* in terms of well-known physical parameters. Combining $\nabla\:\mu\:\left(x,t\right)=\left(\frac{\sigma\:}{D}\right)\frac{RT}{C\left(x,t\right)}\nabla\:\phi\:\left(x,t\right)$ and $\nabla\:\phi\:\left(x,t\right)=\left(\frac{{\phi\:}_{0}}{{P}_{0}}\right)\nabla\:P\left(x,t\right),$ yields an expression of $\nabla\:P\left(x,t\right)$ in terms of $\nabla\:\mu\:(x,t)$: $\nabla\:P(x,t)=\left(\frac{D}{\sigma\:}\right)\left(\frac{1}{RT}\right)\frac{1}{2\pi\:K\left({k}_{0}\right)}C(x,t)\nabla\:\mu\:(x,t).$ Comparing this with Eq. (14) shows that ionic mobility satisfies $b=\frac{D}{RT},$ which matches the well-known Einstein relation^32^. These arguments extend naturally to polarization wave packets involving many $\left(k,\omega\:\right)$ components (Supplementary Information).

### Polarization wave packet in the V1 region and its spreading

As discussed previously, the slowly propagating polarization field *P(x,t)* in the V1 region is more realistically represented not by a single $\left(k,\omega\:\right)$-mode, but by a wave packet composed of multiple modes distributed around the fundamental effective cortical modulation parameters ${\Delta\:}\omega\:$ and ${\Delta\:}k$, as defined in Eqs. (3) and  (4), respectively. For this polarization wave packet, we obtain the following expression (Supplementary Information for derivation):

15 $$P(x,t) = P_{0} e^{{ - i(k_{0} x - \omega _{0} t)}} A\left( t \right){\mathrm{exp}}\left[ { - \frac{{\left( {x - v_{g} t)^{2} } \right.}}{{4(\alpha - i\beta t/2)}}} \right]$$

Here, the amplitude prefactor $A\left(t\right)$ is defined as ${\left\{A\left(t\right)\right\}}^{2}=\left(\frac{\pi\:}{\alpha\:-i\beta\:t/2}\right)$, and $\alpha\:=1/\left(2{\sigma\:}^{2}\right)$, where $\sigma\:$ is the standard deviation of the wave-number distribution. It should be noted that *k*-dependent $\omega\:$ values expressed in Eq. (13) are now smeared in ${v}_{g}$ and $\beta\:$ terms in Eq. (15). Taking the modulus of Eq. (15), the *x*-dependent envelope of the polarization wave packet becomes:

16 $$\:\begin{array}{cccc}&\:\left|P(x,t)\right|={P}_{0}\left|{e}^{-i({k}_{0}x-{\omega\:}_{0}t)}\right|{\left(\frac{{\pi\:}^{2}}{{\alpha\:}^{2}+{\beta\:}^{2}{t}^{2}/4}\right)}^{1/4}\mathrm{e}\mathrm{x}\mathrm{p}\left[-\frac{\alpha\:(x-{v}_{g}t{)}^{2}}{4({\alpha\:}^{2}+{\beta\:}^{2}{t}^{2}/4)}\right]&\:&\:\end{array}$$

Because $\left|{e}^{-i({k}_{0}x-{\omega\:}_{0}t)}\right|=1$, it is retained only to indicate that this wave packet originates from the fundamental modulation wave with the basic frequency ${\omega\:}_{0}={\Delta\:}\omega\:={\omega\:}_{\mathrm{v}\mathrm{p}}$ and the basic wave number ${k}_{0}={\Delta\:}k$. Equation (16) shows that $\left|P(x,t)\right|$ has a Gaussian envelope centered at $x={v}_{g}t$, with a width that gradually increases over time, that is, a dispersive spreading characteristic. In physical terms, Eq. (16) states that the polarization response does not simply translate rigidly along the cortex. Rather, its center moves at the group velocity ${v}_{g}$, while its spatial width increases when $\beta\:\ne\:0$. This broadening reduces the peak amplitude of the wave packet, providing the mathematical basis for the lifetime estimates described in the next paragraph (Eqs. 17 and 18). Importantly, the parameters ${\omega\:}_{0}$ and ${k}_{0}$ in Eqs. (15) and (16) do not refer to the physical optical frequency $\sim\:3.5\times\:{10}^{15}{\hspace{0.17em}}{\mathrm{s}}^{-1}$ or optical wave number $\sim\:{10}^{7}{\hspace{0.17em}}{\mathrm{m}}^{-1}$, but instead correspond to the much smaller modulation parameters ${\Delta\:}\omega\:\approx\:150{\hspace{0.17em}}{\mathrm{s}}^{-1}$ (Eq. 3) and ${\Delta\:}k\approx\:{10}^{4}{\hspace{0.17em}}{\mathrm{m}}^{-1}$ (Eq. 4). Thus, the wave packet *P(x,t)* represents the superposition of polarization modes around this fundamental effective cortical modulation wave, rather than the high-frequency physical optical field itself.

**Fig. 5:**
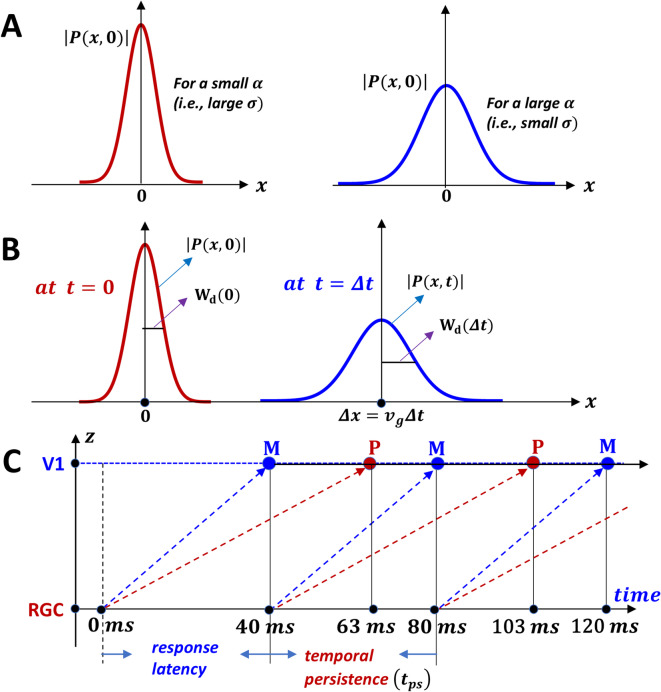
Polarization wave packets and temporal persistence. (**A**) Effect of the standard deviation on the shape of $\left|P\left(x,t\right)\right|$ at a fixed time, *t=0*, where $P\left(x,t\right)$ denotes the polarization response, ${v}_{g}$ is the group velocity of the wave-packet envelope, $\alpha\:=\frac{1}{2{\sigma\:}^{2}}$ is the parameter controlling the initial spectral width, $\beta\:=({d}^{2}\omega\:/d{k}^{2}{)}_{{k}_{0}}$is the dispersion coefficient, and ${t}_{lf}$ is the effective lifetime defined by peak-amplitude decay or envelope broadening. Here, $\sigma\:$ signifies the standard deviation in the wave-number distribution. In other words, *k*-dependent polarization in Gaussian approximation is given by $P\left(k\right)={P}_{o}{e}^{-\alpha\:{\left(k-{k}_{o}\right)}^{2}},$ where $\alpha\:=1/2{\sigma\:}^{2}.$ From Eq. (16), it can be shown immediately that $\left|P\left(x,0\right)\right|={\left(\frac{\pi\:}{\alpha\:}\right)}^{\frac{1}{2}}{P}_{o}\mathrm{exp}\left(-\frac{{x}^{2}}{4\alpha\:}\right).$ It should be noted that the parameter $\alpha\:$ is now in the denominator side of a Gaussian envelope. In contrast, $\alpha\:$ is placed in the numerator side of *k*-dependent Gaussian polarization $P\left(k\right).$ (**B**) Propagation of Gaussian wave packet $\left|P\left(x,t\right)\right|$ with the group velocity ${v}_{g},$ showing spreading behavior owing to non-zero $\beta\:$. Initially, the peak value of $\left|P\left(x,0\right)\right|$ at *x=0* is given by ${\left|P\left(\mathrm{0,0}\right)\right|=\left(\frac{\pi\:}{\alpha\:}\right)}^{\frac{1}{2}}{P}_{o}$ with its HWHM denoted by ${W}_{d}\left(0\right)=\sqrt{4\alpha\: \cdot \:ln2}$. On the contrary, the peak value of $\left|P\left(x,t\right)\right|$ at $t=\varDelta\:t$ is given by $\left|P\left(\varDelta\:x,\varDelta\:t\right)\right|={P}_{o}{\left(\frac{{\pi\:}^{2}}{{\alpha\:}^{2}+\frac{{\beta\:}^{2}({\varDelta\:t)}^{2}}{4}}\right)}^{\frac{1}{4}}\:,$ where $\varDelta\:x={v}_{g}{\Delta\:}t$. Here, it should be noted that $\varDelta\:x$ is not uncertainty in the position, but denotes the peak position at $t=\varDelta\:t.$ It can be shown that ${W}_{d}\left(\varDelta\:t\right)$ is given by the following expression: ${W}_{d}\left(\varDelta\:t\right)=\:{W}_{d}\left(0\right){\left(1+\frac{{\beta\:}^{2}({\varDelta\:t)}^{2}}{4{\alpha\:}^{2}}\right)}^{\frac{1}{2}}\:$ (Supplementary Information for details). **(C)** A schematic diagram that shows the response latency of 40 ms for the M pathway and the temporal persistence (${t}_{ps}$) of the same period of 40 ms. Here, two visual pathways, M and P, are arranged in parallel to the *z*-direction.

Because of dispersive spreading, the height (peak amplitude) of the polarization wave packet decreases, and its width increases over time (Fig. 5). To quantify this, let the peak intensity at time *t=0* and position *x=0* be denoted: ${I}_{\mathrm{0,0}}.$ Similarly, let the peak intensity at time $t={\Delta\:}t$ and position $x={\Delta\:}x$ be ${I}_{{\Delta\:}x,{\Delta\:}t}.$ Using Eq. (16), the ratio of these two intensities becomes:

17 $$\begin{array}{cccc}&\:\frac{{I}_{{\Delta\:}x,{\Delta\:}t}}{{I}_{\mathrm{0,0}}}={\left(\frac{\mid\:P({\Delta\:}x,{\Delta\:}t)\mid\:}{\mid\:P\left(\mathrm{0,0}\right)\mid\:}\right)}^{2}={\left(\frac{{\alpha\:}^{2}}{{\alpha\:}^{2}+{\beta\:}^{2}({\Delta\:}t{)}^{2}/4}\right)}^{1/2}.&\:&\:\end{array}$$

Since ${\Delta\:}x={v}_{g}{\Delta\:}t$, the intensity decays entirely as a function of time. If we define the lifetime of the polarization wave packet as the time at which the peak intensity decays to one-tenth of its initial value, we denote this time as ${t}_{lf\left(p\right)}.$ From Eq. (17), one then obtains: ${t}_{lf\left(p\right)}=\frac{\left(2\sqrt{99}\right)\alpha\:}{\beta\:}\approx\:\frac{20\alpha\:}{\beta\:}.$ Here, $\sigma\:=1/\sqrt{2\alpha\:}$ is the standard deviation of the wave-number distribution; thus, larger $\sigma\:$ (broader *k*-distribution) leads to a shorter lifetime ${t}_{lf\left(p\right)}$. The parameter $\beta\:=({d}^{2}\omega\:/d{k}^{2}{)}_{{k}_{0}}$ is the principal factor controlling the spreading of the wave packet; smaller $\beta\:$ yields a longer lifetime.

In addition to amplitude decay, the lifetime can also be defined through the spreading of the half-width at half-maximum (HWHM) of the Gaussian envelope. Let the HWHM at *t=0* be ${W}_{d}\left(0\right).$ Similarly, let the HWHM at $t={\Delta\:}t$ be ${W}_{d}\left({\Delta\:}t\right).$ Then one obtains the following ratio (Supplementary Information for derivation):

18 $$\begin{array}{cccc}&\:\frac{{W}_{d}\left({\Delta\:}t\right)}{{W}_{d}\left(0\right)}={\left(1+\frac{{\beta\:}^{2}({\Delta\:}t{)}^{2}}{4{\alpha\:}^{2}}\right)}^{1/2}.&\:&\:\end{array}$$

If the lifetime is defined as the time when the HWHM becomes ten times broader than its initial value, one obtains: ${t}_{lf\left(w\right)}=\frac{\left(2\sqrt{99}\right)\alpha\:}{\beta\:}\approx\:\frac{20\alpha\:}{\beta\:}.\:$ The remarkable fact that two distinct definitions of lifetime yield exactly the same functional form ${t}_{lf}=\left(2\sqrt{99}\right)\alpha\:/\beta\:$ highlights the internal mathematical consistency of the spreading behavior of $\left|P(x,t)\right|$ in the V1 region.

## Discussion

### Temporal persistence and lifetime of the polarization wave packet

In visual cognition, response latency refers to the time required for signals from retinal ganglion cells (RGCs) to reach the primary visual cortex (V1). According to the measurements of Schmolesky et al.^23^, the response latency of the M visual pathway in macaque monkeys is 40 ms, whereas the latency of the neighboring P visual pathway is about 63 ms. The latter value lies between the arrival times of successive ion-influx signals through the M pathway at about 40 ms and 80 ms (Fig. 5c). This implies that, between 40 and 80 ms, visual information obtained at time *t=0* from the photoreceptors remains present in the V1 region without being completely extinguished. By the same reasoning, between 80 ms and 120 ms, visual information acquired at *t=40* ms is expected to persist in the V1 region (Fig. 5c). These observations lead to the important conclusion that the temporal persistence of a visual image is determined primarily by the response latency of the M visual pathway, yielding a characteristic value of about 40 ms for macaque monkeys. Although the P pathway exhibits a longer response latency (63 ms), it does not set the temporal persistence; even when referenced to the P pathway, the temporal persistence remains 40 ms, as given by 103–63 ms (Fig. 5c).

Let the temporal persistence be denoted by ${t}_{ps}$. It may then be decomposed into two inter-channel time delays: ${t}_{ps}=\:{\Delta\:}{t}_{M-P}+{\Delta\:}{t}_{P-M}=\left(63-40\right) \; \mathrm{ms}+\left(80-63\right) \; \mathrm{ms}=23 \; \mathrm{ms}+17 \; \mathrm{ms}.$ Here, ${\Delta\:}{t}_{M-P}$represents the delay of ion-influx arrival through the P pathway relative to the neighboring M pathway (23 ms; Fig. 5c), while ${\Delta\:}{t}_{P-M}$ corresponds to the time interval (17 ms) after the P-pathway activation until the arrival of a new ion-influx signal through the next M pathway (Fig. 5c). The delay ${\Delta\:}{t}_{M-P}$ must be greater than or comparable to the effective lifetime of the polarization wave packet *P(x,t)*, ${t}_{lf}\approx\:\frac{20\:{\alpha\:}_{\left(M\right)}}{\beta\:},$ as discussed previously. Thus, the condition ${\Delta\:}{t}_{M-P}=23 \; \mathrm{ms}\ge\:\frac{2{0\:\alpha\:}_{\left(M\right)}}{\beta\:}$ must be satisfied. If this rule is not followed, visual information left over from the M visual pathway would blend with fresh input from the P visual pathway, causing confusion or ambiguity when we perceive things. A similar requirement applies to the delay ${\Delta\:}{t}_{P-M}=17$ms, ${\Delta\:}{t}_{P-M}\ge\:\frac{20\:{\alpha\:}_{\left(P\right)}}{\beta\:},$ where ${\alpha\:}_{\left(P\right)}$ characterizes the polarization wave packet induced by the P-pathway ion influx. Assuming that the dispersion parameter $\beta\:$ is pathway-independent, one obtains the inequality ${\alpha\:}_{\left(M\right)}>{\alpha\:}_{\left(P\right)}$. Finally, it should be noted that the present discussion is based on the experimental data obtained from macaque monkeys^23^. Whether the same quantitative relationships apply directly to human visual cognition remains an open question and requires further experimental validation.

### Modulated waves *versus* cortical travelling waves

As mentioned previously, experimentally observed traveling waves in the visual cortex propagate with velocities in the range of approximately 1.6 ~ 2.2 cm/s^15,16^. These values are slightly higher than the characteristic propagation velocity of the effective cortical modulation wave, ${v}_{m}=\frac{{\Delta\:}{\omega\:}_{\mathrm{e}\mathrm{f}\mathrm{f}}}{{\Delta\:}k}=\frac{{\omega\:}_{vp}}{{\Delta\:}k}\approx\:1.5 \; \mathrm{cm/s}.$ Here, it should be noted that the modulated wave described by Eq. (2), with ${\Delta\:}\omega\:={\omega\:}_{0}$ and ${\Delta\:}k={k}_{0}$, can be regarded as a single $\left({k}_{o},\:{\omega\:}_{o}\right)$-mode wave. The fact that the experimentally observed traveling waves propagate faster than this characteristic single-mode velocity (${v}_{m}=\frac{{\omega\:}_{0}}{{k}_{0}}=1.5 \; \mathrm{cm/s}$) suggests that the cortical traveling waves are not single-mode excitations, but rather polarization wave packets composed of a finite range of wave numbers and frequencies. As described previously, ion-influx signals arriving through a given anatomical visual pathway originate from information integrated over more than one hundred photoreceptors, each sensitive to a range of wavelengths and frequencies^20,21^. It is therefore natural to expect that the resulting traveling activity in V1 reflects a superposition of multiple $(k,\omega\:)$ components and exhibits wave-packet–like behavior. For such a wave packet, the relevant propagation speed is the group velocity, ${v}_{g}={\left(\frac{d\omega\:}{dk}\right)}_{{k}_{0}},$ which generally differs from the phase velocity of the effective cortical modulation wave having a single mode character, ${v}_{m}=\frac{{\omega\:}_{0}}{{k}_{0}}\approx\:1.5\:{\mathrm{cm/s}}.$

The observed enhanced velocity of traveling waves in the visual cortex can also be explained in terms of the damping effect without invoking a multi $\left(k,\omega\:\right)$-mode polarization wave. The damping term ${e}^{-t/\left(2\tau\:\right)},$ which arises from the imaginary part of the angular frequency $\omega\:$ (Eq. 9) through ${e}^{+i\omega\:t}={e}^{+i(+i/2\tau\:)t},$ tends to suppress high-*k* modes since the effective relaxation time $\tau\:$ is inversely proportional to *k* [See Eq. (8) for the definition of $\tau\:$]. This reduced $\tau\:$ values for high-*k* modes enhance the damping effect in $P\left(x,t\right)\:(\propto\:\:{e}^{-t/\left(2\tau\:\right)})$ and then leads to an enhanced propagation velocity as $v=\frac{\omega\:}{k}=\sqrt{\frac{D}{\tau\:}}$ (Eq. 9). The higher propagation velocities reported in awake monkeys^33^ should not be interpreted as contradicting the present estimate. Rather, they indicate that the value ${v}_{m}\approx\:1.5{\hspace{0.17em}}\mathrm{c}\mathrm{m}/\mathrm{s}$ for humans should be regarded as a characteristic scale, not a universal velocity. Within the present framework, species- and state-dependent changes in cortical traveling-wave speed may arise from changes in effective tissue parameters, cortical state, stimulus condition, and the spectral composition of the multi-$\left(k,\omega\:\right)$ wave packet. Thus, the comparison with awake-monkey data is best understood as evidence for variability in cortical wave propagation rather than as a direct test of a single fixed value of ${v}_{m}$.

### Model limitations and experimental outlook

The present work should be interpreted as a theoretical and coarse-grained biophysical model, not as direct experimental demonstration of cortical polarization waves. Several simplifying assumptions were introduced to make the problem analytically and numerically tractable. In particular, the cortical medium was treated as approximately linear, homogeneous, and isotropic, and the scalar-potential dynamics were described using effective parameters such as the diffusion coefficient *D*, relaxation time $\tau\:$, dielectric susceptibility $\chi\:$, and polarization relaxation time ${\tau\:}_{p}$. Real cortical tissue is heterogeneous and anisotropic, with spatial variations in conductivity, permittivity, cellular composition, synaptic density, and ion-channel distribution. Such microstructural variations may modify the local propagation speed, damping rate, and dispersive broadening of the proposed polarization-wave packet.

The velocity ${v}_{m}\approx\:1.5$ cm/s should therefore be regarded as a characteristic scale rather than a universal velocity for all cortical traveling waves. Experimentally measured traveling-wave speeds can vary with species, cortical state, stimulus condition, recording modality, and the operational definition of phase or wavefront propagation. Within the present framework, such variability may arise from changes in the effective ${\Delta\:}\omega\:/{\Delta\:}k$ ratio, the tissue parameters entering *D* and $\tau\:$, and the spectral composition of the multi-$\left(k,\omega\:\right)$ polarization wave packet. Thus, the agreement between the estimated velocity and reported visual-cortical traveling-wave speeds should be regarded as an initial consistency check rather than definitive validation.

Future experimental tests should examine whether slowly propagating cortical activity is accompanied by measurable extracellular-potential gradients, current-source-density patterns, or field responses consistent with polarization-like dynamics. Relevant approaches may include local field potential recordings, current-source-density analysis, voltage-sensitive dye imaging, calcium imaging, EEG/MEG phase-propagation measurements, fNIRS-based cortical activity mapping^34^, and high-resolution MRI-based conductivity or permittivity mapping. Emerging bioelectronic retina/visual-interface technologies^35^ may also provide complementary platforms for controlled visual input and neural response studies. Simultaneous or coordinated measurements of wavefront propagation, amplitude decay, and spatial broadening would be particularly useful for evaluating the predicted dispersive spreading and its possible relation to reduced temporal overlap between neighboring geniculocortical inputs.

## Conclusions

Our systematic investigation into the slowly moving biophysical travelling waves within the visual cortex has led to the following conclusions: **(i)** The modulated wave relevant to visual cognition is characterized by $\varDelta\:\omega\:$ ≈ 150 s⁻¹, ${\Delta\:}k\:$≈ 10⁴ m⁻¹, and ${v}_{m}\:$≈ 1.5 cm/s. Thus, it is not an optical wave, but an effective cortical modulation wave generated after phototransduction and geniculocortical transformation. **(ii)** The velocity of the scalar potential ridge, as obtained by using a telegraph-type model simulation, is about 1.5 cm/s. Notably, this value is equal to the independently predicted velocity of the effective cortical modulation wave (${v}_{m}=$1.5 cm/s). **(iii)** Because of the linear convolution relation, the polarization wave $P\left(x,t\right)$ and the scalar potential field $\phi\:(x,t)$ are kinematically linked, forming an object-shadow relation. Therefore, they propagate with the same velocity. **(iv)** We propose that the observed cortical travelling waves may be modeled as polarization-field responses that are responding to the effective cortical modulation waves, in accordance with the input-response relation in a convolution framework. **(v)** The polarization field in the V1 region contains a distribution of wave numbers. A multi-polarization wave packet disperses over time, which may reduce cross-channel interference and help stabilize visual perception.

## Methods

### Simulations of cortical scalar potentials by telegraph-type equation

We model the extracellular scalar potential $\phi\:\left(x,t\right)$ in an isotropic, homogeneous cortical tissue under quasi-static conditions by a damped telegraph-type PDE derived from the current continuity and the Debye-type polarization relaxation. The relevant PDE is given in Eq. (8), namely, $\tau\:\frac{{\partial\:}^{2}\phi\:}{\partial\:{t}^{2}}+\frac{\partial\:\phi\:}{\partial\:t}=D{\nabla\:}^{2}\phi\:+S\left(x,t\right).$ Here *D* is the effective diffusivity, $\tau\:\:\left[\equiv\:{\tau\:}_{\mathrm{e}\mathrm{f}\mathrm{f}}\left(k\right)\right]$ is the effective relaxation time defined in Eq. (8), and *S(x,t)* is the impressed source term associated with synaptic ionic currents. Baseline parameters were chosen to match the characteristic wave speed $v=\sqrt{D/\tau\:}\approx\:1.5 \; \mathrm{c}\mathrm{m}{\hspace{0.17em}}{\mathrm{s}}^{-1}$ and the visual-persistence timescale $\tau\:\approx\:0.042 \; \mathrm{s}$. Two localized thalamocortical input streams were modeled as separable Gaussian sources in space and causal pulses in time:$\:\:S(x,t)={A}_{1}\mathrm{exp}\left[-\frac{(x-{x}_{1}{)}^{2}}{2{\sigma\:}_{x}^{2}}\right] \cdot p(t-{t}_{1})+{A}_{2}{\hspace{0.17em}}\mathrm{e}\mathrm{x}\mathrm{p}\left[-\frac{(x-{x}_{2}{)}^{2}}{2{\sigma\:}_{x}^{2}}\right]{\hspace{0.17em}}\cdot p(t-{t}_{2}),$ with ${x}_{2}-{x}_{1}={\Delta\:}{x}_{\mathrm{src}}=0.4 \; \mathrm{m}\mathrm{m}$, ${\sigma\:}_{x}=0.15 \; \mathrm{m}\mathrm{m}$. The temporal kernel $p(t-{t}_{j})$ was a brief, causal log-normal–like pulse (rise-and-decay) of the width $w\approx\:8 \; \mathrm{m}\mathrm{s}$, peaking shortly after its onset; the second source was delayed by ${\Delta\:}{t}_{\mathrm{src}}=30 \; \mathrm{m}\mathrm{s}$ to reflect pathway timing differences (M vs. P, synaptic hops). Amplitudes ${A}_{1},\:{A}_{2}\:\:$were equal unless stated.

We used second-order centered differences in space and a damped leap-frog–type update for time: ${\phi\:}_{j}^{n+1}=2{\phi\:}_{j}^{n}-{\phi\:}_{j}^{n-1}+\frac{{\Delta\:}{t}^{2}}{\tau\:}\left(D{\hspace{0.17em}}{\delta\:}_{xx}{\phi\:}_{j}^{n}+{S}_{j}^{n}\right)-\frac{{\Delta\:}t}{\tau\:}\left({\phi\:}_{j}^{n}-{\phi\:}_{j}^{n-1}\right)$ with ${\delta\:}_{xx}{\phi\:}_{j}^{n}={\phi\:}_{j+1}^{n}-2{\phi\:}_{j}^{n}+{\phi\:}_{j-1}^{n}$. Neumann boundaries (zero-flux) were imposed by copying edge-adjacent values at each step. Initial conditions are ${\phi\:}^{0}={\phi\:}^{-1}=0$. We recorded (i) a space–time map of $\phi\:(x,t)$ (down-sampled in *t*) and (ii) spatial snapshots $\phi\:(x,{t}_{k})$ at selected times. The front velocity was determined by applying linear regression to the ridge of the space–time map, and it was accurately recovered to within a few percent. Parameter sweeps varying $\tau\:$ (0.02–0.06 s) and *D* accordingly (for a fixed *v*) confirmed qualitative robustness of damped traveling ridges, with temporal damping scale $\approx\:2\tau\:$ and spatial attenuation $\mathcal{l}\approx\:2\sqrt{D\tau\:}\sim\:1.3 \; \mathrm{m}\mathrm{m}.$ Every simulation was conducted using double precision. The method is grid-convergent (halving ${\Delta\:}x,\:{\Delta\:}t$ changed *v* by < 1%).

### Kernels${K}\left({x}\right)$ and ${K}\left({k},{\omega\:}\right)$ for telegraph-type equation

For a translationally invariant single-mode wave, application of the convolution theorem yields the following equality: $\left(K*{e}^{-i{k}_{0}x}\right)\left(x\right)=K\left({k}_{0}\right){e}^{-i{k}_{0}x}=(2\pi\:{P}_{0}{e}^{+i\omega\:t}{)}^{-1}\phi\:\left(x,t\right),$ as previously shown in Eq. (12). This indicates that any kernel $K\left(x\right)$ is permissible as long as its Fourier transform satisfies $K\left({k}_{0}\right)\ne\:0$. In our case, $K\left({k}_{0}\right)={\phi\:}_{0}/\left(2\pi\:{P}_{0}\right)$ as $\phi\:\left(x,t\right)$ can be represented by the following relation for a single $\left({k}_{o},\omega\:\right)$-mode: $\phi\:\left(x,t\right)={\phi\:}_{0}{e}^{-i\left({k}_{0}x-\omega\:t\right)}$. It is interesting to identify the functional form of $K\left({k}_{0}\right)$ for the telegraph-type equation (8) used to simulate the propagation velocity of the scalar-potential front $\phi\:\left(x,t\right).$ Substituting $\phi\:\left(x,t\right)={\phi\:}_{0}{e}^{-i\left(kx-\omega\:t\right)}$ into Eq. (8) yields the following Helmholtz-type equation for $\phi\:(x,\omega\:)$ in the Fourier $\omega\:$-domain:

19 $$\left({\nabla\:}^{2}+{k}_{eff}^{\:2}\right)\phi\:\left(x,\omega\:\right)=\:-\frac{S\left(x,\omega\:\right)}{D}$$

where ${k}_{eff}^{\:2}=\:\frac{\tau\:{\omega\:}^{2}-i\omega\:}{D}$ and $\phi\:\left(x,\omega\:\right)=\int\:\phi\:\left(x,t\right){e}^{-i\omega\:t}dt.$ Suppose we could find a function $G\left(x\right)$ that solves the above Helmholtz equation with a delta-function ‘source’, namely, $\left({\nabla\:}^{2}+{k}_{eff}^{\:2}\right)G\left(x\right)=\delta\:\left(x\right).$ Then, we could express $\phi\:\left(x,\omega\:\right)$ as an integral: $\phi\:\left(x,\omega\:\right)=\int\:G\left(x-{x}_{o}\right)Q\left({x}_{o}\right)d{x}_{o},$ where $Q\left(x\right)$ does represent the inhomogeneous term, $-\frac{S\left(x,\omega\:\right)}{D}$, in Eq. (19). Substituting this relation into Eq. (19) yields: $\left({\nabla\:}^{2}+{k}_{eff}^{\:2}\right)\:\phi\:\left(x,\omega\:\right)=\int\:\:\left[\left({\nabla\:}^{2}+{k}_{eff}^{\:2}\right)\:G\left(x-{x}_{o}\right)\right]\:Q\left({x}_{o}\right)d{x}_{o}=\int\:\delta\:\left(x-{x}_{o}\right)Q\left({x}_{o}\right)d{x}_{o}=Q\left(x\right).$ The $G\left(x-{x}_{o}\right)$ is thus called the Green function for the Helmholtz equation and represents the ‘response’ to a delta function source since $\left({\nabla\:}^{2}+{k}_{eff}^{\:2}\right)G\left(x\right)=\delta\:\left(x\right)$.

The derivation of the Green function for this standard Helmholtz equation [i.e., $\left({\nabla\:}^{2}+{k}_{eff}^{\:2}\right)\phi\:\left(x,\omega\:\right)=Q\left(x\right)$] requires very lengthy and complicated mathematical manipulations^36^. But the resulting Green function is rather simple^36,37^: $G\left(x\right)=-\frac{{e}^{i{k}_{eff\:}x}}{4\pi\:\:x}.$ The response kernel $K\left(x\right)$ for the telegraph-type equation can be obtained by noting that the inhomogeneous term $Q\left(x\right)$ in the standard Helmholtz equation is replaced by the source term, $-\frac{S\left(x,\omega\:\right)}{D},$ in the Helmholtz equation relevant to the telegraph-type partial differential equation (PDE). Thus, the response kernel for the telegraph-type PDE is $K\left(x\right)=\left(-\frac{1}{D}\right)\left(-\frac{{e}^{i{k}_{eff}x}}{4\pi\:x}\right)=+\frac{{e}^{i{k}_{eff}\:x}}{4\pi\:Dx}$. It is a damped Yukawa/oscillatory-type kernel with the effective diffusion coefficient *D* in the denominator. The corresponding Fourier-space Green function $K\left(k,\omega\:\right)$ can be obtained by carrying out the following four steps: (i) obtain the kernel [$\stackrel{\sim}{K}\left(k,\omega\:\right)={\left(-D{k}^{2}+\tau\:{\omega\:}^{2}-i\omega\:\right)}^{-1}$] by adopting $\phi\:\left(x,t\right)={\phi\:}_{o}{e}^{-i\left(kx-\omega\:t\right)}$ in Eq. (8), (ii) take double Fourier transforms to obtain $\phi\:\left(k,\omega\:\right)=-\stackrel{\sim}{K}\left(k,\omega\:\right)S\left(k,\omega\:\right),$ (iii) show that the source term $S\left(k,\omega\:\right)$ is directly proportional to $P\left(k,\omega\:\right)$ starting from $S\left(x,t\right)=\frac{1}{{C}_{eff}}\left[\nabla\: \cdot \:{\boldsymbol{J}}_{imp}\left(x,t\right)\right]$, and (iv) apply the linear convolution relation in $\left(k,\omega\:\right)$-space, $\phi\:\left(k,\omega\:\right)=K\left(k,\omega\:\right)P\left(k,\omega\:\right),$ to the resulting equation obtained in step (ii). Thus, the kernel in $\left(k,\omega\:\right)$-space is given by $K\left(k,\omega\:\right)=\:\frac{1}{+D{k}^{2}-\tau\:{\omega\:}^{2}+i\omega\:}$.

## Supplementary Information

Below is the link to the electronic supplementary material.

Supplementary Material 1

## Data Availability

All data supporting the findings of this study are available from the corresponding authors upon reasonable request.
