# Supplementary material for: A biophysical model linking cortical scalar potentials and polarization waves to slow traveling activity in vision
Source: Sci Rep. 2026 Jun 9;16:24334. doi: 10.1038/s41598-026-56500-x (PMC13448830; doi:10.1038/s41598-026-56500-x)
Supplement: Supplementary file 1 [file 41598_2026_56500_MOESM1_ESM.pdf]

Supplementary Information

A Biophysical Model Linking Cortical Scalar Potentials and Polarization Waves to Slow Traveling Activity in Vision

Hyun Myung Jang,[1,2]* Youngwoo Jang,[3] and Hyeon Han[4,5,6]*

[1]Research Institute of Advanced Materials, Seoul National University, Seoul 08826, Republic of Korea.

[2](Permanent Address) Pohang University of Science and Technology (POSTECH), Pohang 37673, Republic of Korea.

[3]Department of Cardiology, Gil Medical Center, Gachon University College of Medicine, Incheon 21565, Republic of Korea.

[4]Department of Materials Science and Engineering, Pohang University of Science and Technology (POSTECH), Pohang 37673, Republic of Korea.

[5]Korea-Max Planck-PSI Center for Quantum Emergent Spintronics (KOMQUEST), Pohang 37673, Republic of Korea.

[6]Max Planck Institute of Microstructure Physics, Weinberg 2, Halle (Saale) 06120, Germany.

* Corresponding Authors: **Hyun M. Jang** (email: hmjang@postech.ac.kr) and **Hyeon Han** (email: hyeonhan@postech.ac.kr).

Supplementary Information includes:

(i) Derivation of telegraph-type equation for cortical scalar potentials

(ii) Polarization wave packet and its dispersive spreading in time

(iii) Relation between $\nabla P(x,t)$ and $\nabla C(x,t)$ for a polarization wave packet

(i) Derivation of telegraph-type equation for cortical scalar potentials

In an isotropic, homogeneous cortical medium operating in the low-frequency regime, where the high-frequency component of the displacement current can be neglected, the divergence of the total current ($\nabla \cdot \boldsymbol{J}_{tot}$) vanishes, and the total current consists of four contributions: (i) conduction current, (ii) displacement current, (iii) polarization current, and (iv) impressed ionic current generated by neural activity. Accordingly, we write

$$\nabla \cdot \boldsymbol{J}_{tot} = 0, \quad \boldsymbol{J}_{tot} = \sigma \boldsymbol{E} + \varepsilon \frac{\partial \boldsymbol{E}}{\partial t} + \frac{\partial \boldsymbol{P}}{\partial t} + \boldsymbol{J}_{imp} \tag{S1}$$

where, σ is the tissue conductivity, ε the dielectric permittivity, $\boldsymbol{P}$ the polarization density, and $\boldsymbol{J}_{imp}$ the impressed ionic current. The electric field ($\boldsymbol{E}$) is given by

$$\boldsymbol{E} = -\nabla \phi \tag{S2}$$

Using Eq. (S2), the requirement of $\nabla \cdot \boldsymbol{J}_{tot} = 0$ in Eq. (S1) can be re-written as

$$-\sigma \nabla^2 \phi - \varepsilon \frac{\partial}{\partial t}(\nabla^2 \phi) + \frac{\partial}{\partial t}(\boldsymbol{\nabla} \cdot \boldsymbol{P}) + (\boldsymbol{\nabla} \cdot \boldsymbol{J}_{imp}) = 0 \tag{S3}$$

where $\boldsymbol{P}$ denotes the dielectric polarization which is defined as the dipole moment per unit volume.

Since the dipolar polarization in cortical tissue responds at low frequency, we adopt a Debye-type dielectric relaxation mechanism. Thus, we write

$$\tau_p \frac{\partial \boldsymbol{P}}{\partial t} + \boldsymbol{P} = \varepsilon_0 \chi \mathbf{E} = -\varepsilon_0 \chi \nabla \phi \tag{S4}$$

where χ is the effective dielectric susceptibility in the neuronal layer, $\varepsilon_0 = 8.854 \times 10^{-12} J^{-1} C^2 m^{-1}$, the permittivity of free space, and τ_p the dipole-alignment relaxation time. Taking (i) time-derivative of the above equation and (ii) the divergence of the resulting equation, we obtain

$$\tau_p \frac{\partial^2}{\partial t^2}(\boldsymbol{\nabla} \cdot \boldsymbol{P}) + \frac{\partial}{\partial t}(\boldsymbol{\nabla} \cdot \boldsymbol{P}) = -\varepsilon_p \frac{\partial}{\partial t}(\nabla^2 \phi) \tag{S5}$$

where ε_p denotes $\varepsilon_0 \chi$. Then, combining Eq. (S5) with Eq. (S3) to eliminate $\frac{\partial}{\partial t}(\boldsymbol{\nabla} \cdot \boldsymbol{P})$-term yields

$$-\sigma \nabla^2 \phi - \varepsilon \frac{\partial}{\partial t}(\nabla^2 \phi) - \tau_p \frac{\partial^2}{\partial t^2}(\boldsymbol{\nabla} \cdot \boldsymbol{P}) - \varepsilon_p \frac{\partial}{\partial t}(\nabla^2 \phi) + (\boldsymbol{\nabla} \cdot \boldsymbol{J}_{imp}) = 0 \tag{S6}$$

Under the single-mode *ansatz*, $P(x,t) = P_o\, e^{-i(kx-\omega t)} e^{-i\delta}$, where δ denotes the phase lag or retardation. Thus, $\tan \delta = \chi"(\omega)/\chi'(\omega)$, where $\chi"(\omega)$ denotes the imaginary part of the ω-dependent dielectric susceptibility. Since the dipolar

polarization in cortical tissue responds at low frequency, $e^{-i\delta} \approx e^{o} = 1$. Therefore, we write the following expression for the time-dependent dielectric polarization:

$$P(x,t) = P_o e^{-i(kx-\omega t)} = P(t)e^{-ikx} \quad \text{(S7)}$$

where $P(t) = P_o e^{+i\omega t}$. Similarly, we write

$$\phi(x,t) = \phi_o e^{-i(kx-\omega t)} = \phi(t)e^{-ikx} \quad \text{(S8)}$$

where $\phi(t) = \phi_o e^{+i\omega t}$. In cortical medium, the dielectric permittivity ε can be written as $\varepsilon_o\varepsilon_r$, where ε_r denotes the relative dielectric permittivity (*i.e.*, dielectric constant). Thus, we write $\varepsilon = \varepsilon_o\varepsilon_r = \varepsilon_o(1+\chi)$. If we assume that χ in cortical medium is substantially bigger than 1, we have the following approximation: $\varepsilon \approx \varepsilon_0\chi$. Since ε_p is defined as $\varepsilon_0\chi$, we have the following approximation: $\varepsilon \approx \varepsilon_p = \varepsilon_0\chi$. Substituting this expression of ε_p into Eq. (S6), we obtain the following partial differential equation (PDE):

$$-\sigma\nabla^2\phi - 2\varepsilon\frac{\partial}{\partial t}(\nabla^2\phi) - \tau_p\frac{\partial^2}{\partial t^2}(\boldsymbol{\nabla}\cdot\boldsymbol{P}) + \left(\boldsymbol{\nabla}\cdot\boldsymbol{J}_{imp}\right) = 0 \quad \text{(S9)}$$

where $\varepsilon \approx \varepsilon_p = \varepsilon_0\chi$ and χ is the dielectric susceptibility in the neuronal layer.

We now eliminate $\frac{\partial^2}{\partial t^2}(\boldsymbol{\nabla}\cdot\boldsymbol{P})$ term in Eq. (S9) and express this in terms of $\frac{\partial^2}{\partial t^2}\phi(x,t)$. To do this, we first compute the divergence term, $\boldsymbol{\nabla}\cdot\boldsymbol{P}$, using Eq. (S7): $\boldsymbol{\nabla}\cdot\boldsymbol{P} = \frac{\partial}{\partial x}P(x,t) + \frac{\partial}{\partial y}P(x,t) + \frac{\partial}{\partial z}P(x,t)$. Since $\frac{\partial}{\partial y}P(x,t) = \frac{\partial}{\partial z}P(x,t) = 0$, $\boldsymbol{\nabla}\cdot\boldsymbol{P} = \frac{\partial}{\partial x}P(x,t) = -kP(x,t)$. Using this expression, we calculate $\frac{\partial^2}{\partial t^2}(\boldsymbol{\nabla}\cdot\boldsymbol{P})$ term to yield

$$\frac{\partial^2}{\partial t^2}(\boldsymbol{\nabla}\cdot\boldsymbol{P}) = (-k)(+i\omega)^2 P_o e^{-i(kx-\omega t)} = +k\omega^2 P_o e^{-i(kx-\omega t)} \quad \text{(S10)}$$

Similarly, we obtain

$$\frac{\partial^2}{\partial t^2}\phi(x,t) = -\omega^2\phi_o e^{-i(kx-\omega t)} \quad \text{(S11)}$$

Combining Eq. (S10) with Eq. (S11) immediately yields

$$\frac{\partial^2}{\partial t^2}(\boldsymbol{\nabla}\cdot\boldsymbol{P}) = -k\left(\frac{P_o}{\phi_o}\right)\frac{\partial^2}{\partial t^2}\phi(x,t) \quad \text{(S12)}$$

Similarly, $\frac{\partial}{\partial t}(\nabla^2\phi)$ term in Eq. (S9) can be rewritten as

$$\frac{\partial}{\partial t}(\nabla^2\phi) = -k^2\frac{\partial\phi}{\partial t} \quad \text{(S13)}$$

Substituting Eq. (S12) and Eq. (S13) into Eq. (S9) and rearranging yield

$$\tau_p k\left(\frac{P_o}{\phi_o}\right)\frac{\partial^2\phi}{\partial t^2} + 2\varepsilon_0\chi k^2\frac{\partial\phi}{\partial t} = +\sigma\nabla^2\phi - \boldsymbol{\nabla}\cdot\boldsymbol{J}_{imp} \quad \text{(S14)}$$

Dividing both sides of Eq. (S14) by $2\varepsilon_0\chi k^2$, we obtain

$$\tau\frac{\partial^2\phi}{\partial t^2}+\frac{\partial\phi}{\partial t}=D\nabla^2\phi+S(x,t) \tag{S15}$$

This is the telegraph-type PDE (Eq. (8) of the main manuscript) used in the simulation of the space-time map of the cortical scalar potential field $\phi(x,t)$. τ appeared in Eq. (S15) is called the effective relaxation time $[\tau\equiv\tau_{eff}(k)]$. D denotes the effective diffusion coefficient in the cortical medium, and $S(x,t)$ is called the source term. They are defined as

$$\tau\equiv\tau_{eff}(k)=\frac{\tau_p\left(\frac{P_o}{\phi_o}\right)}{2\varepsilon_0\chi k}=\frac{k\tau_p\left(\frac{P_o}{\phi_o}\right)}{C_{eff}} \tag{S16}$$

$$D=\frac{\sigma}{2\varepsilon_0\chi k^2}=\frac{\sigma}{C_{eff}} \tag{S17}$$

And

$$S(x,t)=\frac{|\nabla\cdot\boldsymbol{J}_{imp}|}{2\varepsilon_0\chi k^2}=\frac{|\nabla\cdot\boldsymbol{J}_{imp}|}{C_{eff}} \tag{S18}$$

where C_{eff} is the effective capacitance factor and is defined as $2\varepsilon_0\chi k^2$.

(ii) Polarization wave packet and its dispersive spreading in time

The propagating polarization field in the V1 region can be represented by a wave packet $P(x,t)$ composed of multiple (k,ω)-modes distributed around the cognitively fundamental modulation parameters (k_o,ω_o). We thus write $P(x,t)$ by the following integral form using the k-dependent polarization $P(k)$:

$$P(x,t)=\int dk\,P(k)\,e^{-i\{kx-\omega(k)t\}} \tag{S19}$$

If we assume a Gaussian normal distribution, $P(k)$ can be written as

$$P(k)=P_oe^{-\alpha(k-k_o)^2} \tag{S20}$$

where $P_o\equiv P(k_o)$ and $\alpha\equiv 1/2\sigma^2$ with σ defined as the standard deviation in the distribution of wave numbers (ks). In Eq. (S19), $\omega(k)$ denotes k-dependent angular frequency. It is described by Eq. (13) of the main manuscript. Substituting Eq. (S20) and Eq. (13) into Eq. (S19) yields

$$P(x,t)=P_oe^{-i\{k_ox-\omega_ot\}}\int_{-\infty}^{+\infty}dk'\,e^{-ik'(x-v_gt)}e^{-\left(\alpha-\frac{i\beta t}{2}\right)k'^2} \tag{S21}$$

where k' is defined as $k'\equiv k-k_o$. On the other hand, we obtain the following expression for $P(x,0)$ from Eq. (S19):

$$P(x,0) \equiv P(x) = \int_{-\infty}^{+\infty} dk\, P(k) e^{-ikx} \tag{S22}$$

Substituting Eq. (S20) into Eq. (S22) yields

$$P(x) = P_o e^{-ik_o x} \int_{-\infty}^{+\infty} dk' \, exp\left[-\alpha\left\{k' + \left(\frac{ix}{2\alpha}\right)\right\}^2\right] exp\left(-\frac{x^2}{4\alpha}\right) \tag{S23}$$

It is known that $\int_{-\infty}^{+\infty} dk\, e^{-\alpha k^2} = \sqrt{\frac{\pi}{\alpha}}$. Using this equality, Eq. (S23) becomes

$$P(x) \equiv P(x,0) = \sqrt{\frac{\pi}{\alpha}} P_o e^{-ik_o x} e^{-x^2/4\alpha} \tag{S24}$$

Thus, we have

$$|P(x,0)| = \sqrt{\frac{\pi}{\alpha}} P_o e^{-x^2/4\alpha} \tag{S25}$$

Eq. (S24) can be rewritten as

$$P(x) = \sqrt{\frac{\pi}{\alpha}} P_o e^{-ik_o x} e^{-x^2/4\alpha} = P_o e^{-ik_o x} \int_{-\infty}^{+\infty} dk' e^{-ik' x}\, e^{-\alpha k'^2} \tag{S26}$$

By carefully comparing the second expression of Eq. (S26) with Eq. (S21) we deduce the following mathematical manipulations to obtain the final form of $P(x,t)$ from the first expression of Eq. (S26): (i) substitution of '$x - v_g t$' for 'x' term appeared in $e^{-x^2/4\alpha}$ of Eq. (S26) and (ii) substitution of '$\alpha - \frac{i\beta t}{2}$' for '$\alpha$' term appeared in $(\pi/\alpha)^{1/2}$ and $e^{-x^2/4\alpha}$ of Eq. (S26). Thus, we obtain

$$P(x,t) = P_o e^{-i\{k_o x - \omega_o t\}} \left(\frac{\pi}{\alpha - \frac{i\beta t}{2}}\right)^{1/2} exp\left\{-\frac{(x - v_g t)^2}{4\left(\alpha - \frac{i\beta t}{2}\right)}\right\} \tag{S27}$$

This is the final mathematical expression of the polarization wave packet propagating with the group velocity of v_g in V1. From Eq. (S27), $P(x,0) = P_o \left(\frac{\pi}{\alpha}\right)^{1/2} e^{-ik_o x}$ at $t = 0$. Thus, $P(x,0)$ forms a sinusoidal wave with the effective wavelength of $\lambda_0 = 2\pi/k_0$. To obtain the envelope $|P(x,t)|$ we should evaluate the magnitude of the following two terms that appeared in Eq. (S27):

$$\left|\left(\frac{\pi}{\alpha - \frac{i\beta t}{2}}\right)^{1/2}\right|^2 = \left(\frac{\pi}{\alpha - \frac{i\beta t}{2}}\right)^{1/2} \left\{\left(\frac{\pi}{\alpha - \frac{i\beta t}{2}}\right)^{1/2}\right\}^* = \pi^{1/2} \left\{\frac{\left(\alpha + \frac{i\beta t}{2}\right)}{\left(\alpha^2 + \frac{\beta^2 t^2}{4}\right)}\right\}^{1/2} \pi^{1/2} \left\{\frac{\left(\alpha - + \frac{i\beta t}{2}\right)}{\left(\alpha^2 + \frac{\beta^2 t^2}{4}\right)}\right\}^{1/2} \tag{S28}$$

$$\left|exp\left\{-\frac{(x - v_g t)^2}{4\left(\alpha - \frac{i\beta t}{2}\right)}\right\}\right|^2 = exp\left\{-\frac{\alpha (x - v_g t)^2}{2\left(\alpha^2 + \frac{\beta^2 t^2}{4}\right)}\right\} \tag{S29}$$

We obtain the following equation for the x-dependent envelope of the polarization wave packet by substituting these two relations into Eq. (S27):

$$|P(x,t)| = P_0\left|e^{-i(k_0x-\omega_0t)}\right|\left(\frac{\pi^2}{\alpha^2+\beta^2t^2/4}\right)^{1/4}\exp\left[-\frac{\alpha(x-v_gt)^2}{4(\alpha^2+\beta^2t^2/4)}\right] \quad \text{(S30)}$$

where $P_0 = P(k_o)$ and $\left|e^{-i(k_0x-\omega_0t)}\right| = 1$.

We now find the propagation velocity of $\phi(x,t)$ ridge. For this, we first consider the k-dependent scalar potential field under the condition of Gaussian normal distribution

$$\phi(k) = \phi_o e^{-\alpha(k-k_o)^2} \quad \text{(S31)}$$

Thus, $\phi(k,t)$ can be written as

$$\phi(k,t) = \phi(k)e^{+i\omega(k)t} = \phi_o e^{-\alpha(k-k_o)^2}e^{+i\omega(k)t} \quad \text{(S32)}$$

Similarly, $P(k,t)$ can be written using Eq. (S20) as

$$P(k,t) = P(k)e^{+i\omega(k)t} = P_o e^{-\alpha(k-k_o)^2}e^{+i\omega(k)t} \quad \text{(S33)}$$

Performing inverse Fourier transform, we get: $P(x,t) = \int dk\, P(k,t)\, e^{-ikx} = \int dk\, P(k)e^{-i\{kx-\omega(k)t\}}$. As expected, this is equivalent to Eq. (S19) presented previously. Under the single-mode ansatz, $P(x,t) = P_o e^{-i\{k_ox-\omega_ot\}}$. Then, $P(k,t)$ can be written using forward Fourier transform of $P(x,t)$, namely,

$$P(k,t) \equiv \int P(x,t)e^{+ikx}dx = P_o e^{+i\omega_ot}\int e^{-i(k_o-k)x}dx = 2\pi\, P_o e^{+i\omega_ot}\delta(k_o-k) \quad \text{(S34)}$$

The last expression of Eq. (S34) was obtained by applying the following equality: $2\pi\,\delta(k_o-k) = \int_{-\infty}^{+\infty} e^{-i(k_o-k)x}dx$. We have the linear convolution relation in k-space:

$$\phi(k,t) = K(k)P(k,t) \quad \text{(S35)}$$

This is Eq. (11) of the main manuscript. In the below, we will obtain this convolution relation starting from the convolution integral in (x,t)-space. Substituting Eq. (S34) into Eq. (S35) for $P(k,t)$ gives $\phi(k,t)$. Taking inverse Fourier transform of $\phi(k,t)$ then yields $\phi(x,t)$, namely,

$$\phi(x,t) = \int \phi(k,t)e^{-ikx}dk = 2\pi P_o e^{+i\omega_ot}\int K(k)\,\delta(k_o-k)e^{-ikx}dk =$$
$$2\pi P_o e^{+i\omega_ot}K(k_o)e^{-ik_ox} \equiv \phi_o e^{-i(k_ox-\omega_ot)} \quad \text{(S36)}$$

Thus, we deduce $K(k_o)$ from Eq. (S36)

$$K(k_o) = \frac{\phi_o}{(2\pi P_o)} \neq 0 \quad \text{(S37)}$$

Thus, $K(k)$ is a non-zero constant. On the other hand, $K(x)$ is given by $K(x) = \int K(k)e^{-ikx}\,dk = K(k)\,2\pi\delta(x)$. The last expression was obtained by noting that

$\int e^{-ikx}\, dk = 2\pi\delta(x)$. Thus, $K(x) = 2\pi\, K(k) = \frac{\phi_o}{P_o} \neq 0$ only when $x = 0$. Otherwise, $K(x) = 0$. According to the convolution relation, $\phi(x,t)$ can be written as

$$\phi(x,t) = \int K(x-x')P(x',t)\, dx' = \int K\big((x-vt)-x'\big)\, P(x',t)\, dx'$$

$$= (K * P)(x-vt) \qquad \text{(S38)}$$

The second expression of the convolution integral is written for the traveling polarization wave with the velocity v. Accordingly, $P(x,t) = P(x-vt)$. Eq. (38) expresses the velocity preservation. Here, the kernel K does not alter the propagation speed of the traveling polarization wave. Thus, $\phi(x,t)$ inherits the same propagation velocity as $P(x,t)$. The kernel $K(x-x')$ gives a non-zero contribution of (ϕ_o/P_o) only when $x' = x$. In other words, we establish

$$\phi(x,t) = K(0)P(x,t) = \left(\frac{\phi_o}{P_o}\right) P(x,t) \qquad \text{(S39)}$$

Applying forward Fourier transform to Eq. (S39), we obtain the linear convolution relation in k-space, $\phi(k,t) = K(k)P(k,t)$ [Eq. (S35)]: $\phi(k,t) = \int \phi(x,t)\, e^{+ikx} dx = \int K(x-x') e^{+ik(x-x')}\, dx \int P(x',t) e^{+ikx'}\, dx' = K(k)P(k,t)$.

According to Eq. (S37), a non-zero value of $K(k)$ exists, which indicates that the propagation velocity of $\phi(x,t)$ ridge is equal to the group velocity of $P(x,t)$ as we deduce this equal speed from the velocity preservation in Eq. (S38). We now find the propagation velocity of $\phi(x,t)$ ridge to examine whether this prediction holds for $\phi(x,t)$ having a wave-packet form. To do this, we firstly compute $\phi(x,t)$ by applying inverse Fourier transform of $\phi(k,t)$ and Eq. (S32), namely,

$$\phi(x,t) = \int dk\, \phi(k,t)\, e^{-ikx} = \int dk\, \phi(k)\, e^{-i\{kx-\omega(k)t\}} \qquad \text{(S40)}$$

We can obtain the following expression of $|\phi(x,t)|$ by repeatedly applying the procedures between Eq, (S19) and Eq. (S30) to the above expression of $\phi(x,t)$.

$$|\phi(x,t)| = \phi(k_o)\left(\frac{\pi^2}{\alpha^2+\beta^2 t^2/4}\right)^{1/4} \exp\left[-\frac{\alpha(x-v_g t)^2}{4(\alpha^2+\beta^2 t^2/4)}\right] \qquad \text{(S41)}$$

According to Eq. (S41), the propagation velocity of wave-packet-like $\phi(x,t)$ ridge is equal to $v_g\,(=\frac{x}{t})$ because the exponential term in Eq. (S41) should be 1 at the $\phi(x,t)$ ridge. Thus, $(x-v_g t)^2$ appeared in the numerator of exponential term should be 0, showing that the propagation velocity of $\phi(x,t)$ ridge is equal to the group velocity of $P(x,t)$.

Because of dispersive spreading, the peak amplitude of the polarization wave packet decreases, and its width increases over time (Fig. 4B). To quantify this, let the peak intensity at time $t = 0$ and position $x = 0$ be denoted: $I_{0,0}$. Similarly, let the peak intensity at time $t = \Delta t$ and position $x = \Delta x$ be $I_{\Delta x,\Delta t}$. Using Eq. (S30), the ratio of these two intensities becomes:

$$\frac{I_{\Delta x,\Delta t}}{I_{0,0}} = \left(\frac{|P(\Delta x,\Delta t)|}{|P(0,0)|}\right)^2 = \left(\frac{\alpha^2}{\alpha^2+\beta^2(\Delta t)^2/4}\right)^{1/2} \tag{S42}$$

Since $\Delta x = v_g \Delta t$, the intensity decays entirely as a function of time. If we define the lifetime of the polarization wave packet as the time at which the peak intensity decays to one-tenth of its initial value, we denote this time as $t_{lf(p)}$. From Eq. (S42), we then obtain

$$t_{lf(p)} = \frac{(2\sqrt{99})\alpha}{\beta} \approx \frac{20\alpha}{\beta} \tag{S43}$$

Here, $\sigma = 1/\sqrt{2\alpha}$ is the standard deviation of the wave-number distribution; thus, larger σ (broader k-distribution) leads to a shorter lifetime $t_{lf(p)}$. The parameter $\beta = (d^2\omega/dk^2)_{k_0}$ is the principal factor controlling the spreading of the wave packet; smaller β yields a longer lifetime.

In addition to amplitude decay, the lifetime can also be defined through the spreading of the half-width at half-maximum (HWHM) of the Gaussian envelope. Let the HWHM at $t = 0$ be $W_d(0)$. Similarly, let the HWHM at $t = \Delta t$ be $W_d(\Delta t)$. From Eq. (S30), we obtain the following expression for $|P(x,t)|$ at $t = 0$:

$$|P(x,0)| = P_o\left(\frac{\pi}{\alpha}\right)^{1/2} e^{-x^2/4\alpha} \tag{S44}$$

We then consider the time-dependent HWHM to show the degree of spreading in time. According to the definition of HWHM, we have the following requirement at $t = 0$:

$$|\,P(x_{HM},0)| = \frac{1}{2}|P(0,0)| \tag{S45}$$

where x_{HM} denotes the position of HWHM at $t = 0$. Thus, $x_{HM} = W_d(0)$. Combining Eq. (S45) with Eq. (S44) yields

$$W_d(t=0) = W_d(0) = x_{HM} = \sqrt{4\alpha\, ln2} \tag{S46}$$

The peak position of $P(x,t)$ at time $t = \Delta t$ is $\Delta x = v_g \Delta t$. Thus, we have the following expression for $|P(\Delta x,\Delta t)|$ from Eq. (S30):

$$|P(\Delta x, \Delta t)| = P_0\left(\frac{\pi^2}{\alpha^2+\beta^2(\Delta t)^2/4}\right)^{1/4} \tag{S47}$$

On the contrary, we have the following expression of $|P(x_{HM}, \Delta t)|$ at $x = x_{HM}$ from Eq. (S30):

$$|P(x_{HM}, \Delta t)| = P_0\left(\frac{\pi^2}{\alpha^2+\beta^2(\Delta t)^2/4}\right)^{1/4} \exp\left[-\frac{\alpha(x_{HM}-v_g\Delta t)^2}{4\{\alpha^2+\frac{\beta^2(\Delta t)^2}{4}\}}\right] \tag{S48}$$

According to the definition of HWHM, we establish the following equality:

$$\frac{1}{2}|P(\Delta x, \Delta t)| = |P(x_{HM}, \Delta t)| \tag{S49}$$

Substituting Eq. (S47) and Eq. (S48) into the equality requirement given in Eq. (S49) yields the following relation at $x = x_{HM}$:

$$4\left(\alpha^2 + \frac{\beta^2(\Delta t)^2}{4}\right) ln2 = \alpha\left(x_{HM} - v_g\Delta t\right)^2 \tag{S50}$$

On the other hand, as shown in the $|P(x,t)|$ profile presented in **Figure S1**, we have the following expression for x_{HM} at $t = \Delta t$:

$$x_{HM} = HWHM + \Delta x = W_d(\Delta t) + v_g\Delta t \tag{S51}$$

Substituting Eq. (S51) into Eq. (S50) and comparing this with Eq. (S46) yields

$$\frac{W_d(\Delta t)}{W_d(0)} = \left(1+\frac{\beta^2(\Delta t)^2}{4\alpha^2}\right)^{1/2} \tag{S52}$$

This is Eq. (18) of the main manuscript. If the lifetime is defined as the time when the HWHM (i.e., W_d) becomes ten times broader than its initial value, one obtains: $t_{lf(w)} = \frac{(2\sqrt{99})\alpha}{\beta} \approx \frac{20\alpha}{\beta}$. The remarkable fact that two distinct definitions of lifetime yield exactly the same functional form $t_{lf} = \left(2\sqrt{99}\right)\alpha/\beta$ highlights the internal mathematical consistency of the spreading behavior of $|P(x,t)|$ in the V1 region.

We can also deduce the uncertainty relation by examining two profiles, namely, (i) k-dependent polarization profile $P(k)$ and (ii) the polarization wave packet $P(x,t)$ before undergoing the dispersive spreading due to non-zero β. If we take the uncertainty in the wave number Δk as the HWHM of the $P(k)$ profile, we then establish the following equality from Eq. (S20): $P_o e^{-\alpha(k_o+\Delta k-k_o)^2} = \frac{1}{2} P_o e^{-0}$. This immediately yields the following expression for the uncertainty in the wave number Δk:

$$\Delta k = \sqrt{\frac{\ln 2}{\alpha}} \tag{S53}$$

As shown in Eq. (46), the uncertainty (Δx) in the position of the polarization wave packet $P(x,t)$ at $t = 0$ is $\Delta x\,(= x_{HM}) = \sqrt{4\alpha\, ln2}$. Thus, we obtain the following $(\Delta k - \Delta x)$ uncertainty product:

$$\hbar\, \Delta k \Delta x = 2ln2\, \hbar = 1.38\, \hbar\, \geq \frac{1}{2}\hbar \tag{S54}$$

Therefore, Heisenberg's uncertainty relation also holds for the polarization wave packet in the V1 region.

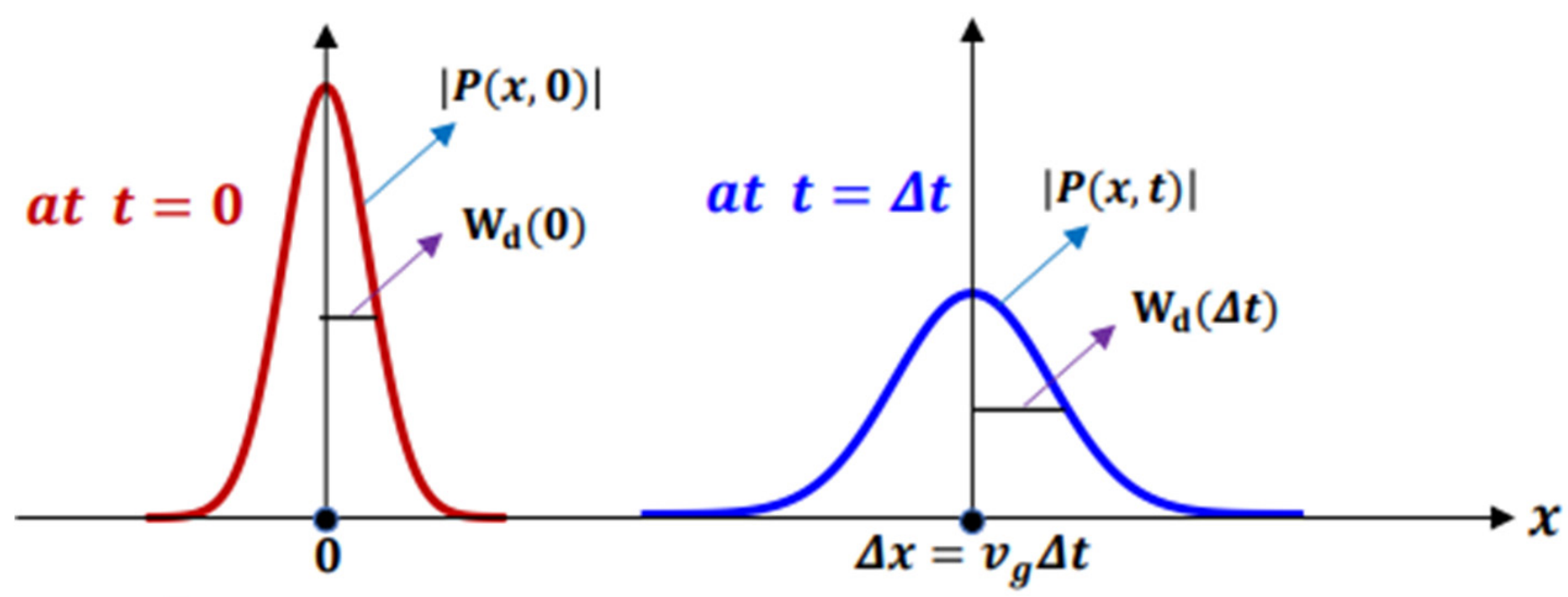

Figure S1. Propagation of the polarization wave packet $|P(x,t)|$ with the group velocity v_g, showing spreading behavior owing to non-zero β. Initially, the peak value of $|P(x,0)|$ at $x = 0$ is given by $|P(0,0)| = \left(\frac{\pi}{\alpha}\right)^{\frac{1}{2}} P_o$ with its HWHM denoted by $W_d(0) = \sqrt{4\alpha \cdot ln2}$. On the contrary, the peak value of $|P(x,t)|$ at $t = \Delta t$ is given by $|P(\Delta x, \Delta t)| = P_o \left(\frac{\pi^2}{\alpha^2 + \frac{\beta^2 (\Delta t)^2}{4}}\right)^{\frac{1}{4}}$, where $\Delta x = v_g \Delta t$. As shown in Eq. (S52), $W_d(\Delta t)$ is given by the following expression of the dispersive spreading: $W_d(\Delta t) = W_d(0)\left(1 + \frac{\beta^2 (\Delta t)^2}{4\alpha^2}\right)^{\frac{1}{2}}$.

(iii) Relation between $\nabla P(x,t)$ and $\nabla C(x,t)$ for a polarization wave packet

In Eq. (15) of the main manuscript, we showed the following expression for a polarization wave packet:

$$P(x,t) = P(k_0)\, e^{-i(k_0 x - \omega_0 t)} A(t) \exp\left[-\frac{(x - v_g t)^2}{4\left(\alpha - \frac{i\beta t}{2}\right)}\right] \tag{15}$$

The corresponding scalar potential field can be expressed as

$$\phi(x,t) = \phi(k_0)e^{-i(k_0x-\omega_0t)}A(t)\exp\left[-\frac{(x-v_gt)^2}{4\left(\alpha-\frac{i\beta t}{2}\right)}\right] \quad \text{(S55)}$$

We can readily find the following expression of $\nabla P(x,t)$ from Eq. (15):

$$\nabla P(x,t) = P(k_0)e^{-i(k_0x-\omega_0t)}A(t)\exp\left[-\frac{(x-v_gt)^2}{4\left(\alpha-\frac{i\beta t}{2}\right)}\right]\left\{(-ik_o)-\frac{(x-v_gt)}{2\left(\alpha-\frac{i\beta t}{2}\right)}\right\} \quad \text{(S56)}$$

Similarly, we have the following expression of $\nabla\phi(x,t)$ from Eq. (S55):

$$\nabla\phi(x,t) = \phi(k_0)e^{-i(k_0x-\omega_0t)}A(t)\exp\left[-\frac{(x-v_gt)^2}{4\left(\alpha-\frac{i\beta t}{2}\right)}\right]\left\{(-ik_o)-\frac{(x-v_gt)}{2\left(\alpha-\frac{i\beta t}{2}\right)}\right\} \quad \text{(S57)}$$

We thus have the following ratio from Eqs. (S56) and (S57):

$$\frac{\nabla P(x,t)}{\nabla\phi(x,t)} = \frac{P(k_o)}{\phi(k_o)} \equiv \frac{P_o}{\phi_o} = \frac{1}{2\pi\, K(k_o)} \quad \text{(S58)}$$

The last expression of Eq. (S58) is obtained from Eq. (S37).

We have the following equality from Fick's law and Ohm's law:

$$\boldsymbol{J}_{con} = -D\nabla C(x,t) = \sigma\boldsymbol{E} = -\sigma\nabla\phi(x,t) \quad \text{(S59)}$$

This macroscopic phenomenological relation is valid, regardless of the number of (k,ω) components in a system. Combining Eq. (S58) with Eq. (S59) to eliminate $\nabla\phi(x,t)$ term yields

$$\nabla P(x,t) = \frac{1}{2\pi\, K(k_0)}\left(\frac{D}{\sigma}\right)\nabla C(x,t) \quad \text{(S60)}$$

Eq. (S60) is equal to the first expression of Eq. (14) of the main manuscript. As noted in the main manuscript, Fick's first law in Eq. (S59) can be rewritten in terms of the chemical potential gradient as

$$D\nabla C(x,t) = -\boldsymbol{J}_{con} = +bC(x,t)\nabla\mu(x,t) \quad \text{(S61)}$$

This macroscopically valid relation also holds for a multi-(k,ω) components system. Substituting Eq. (S61) into Eq. (S60) yields

$$\nabla P(x,t) = \frac{1}{2\pi\, K(k_0)}\left(\frac{b}{\sigma}\right)C(x,t)\,\nabla\mu(x,t) \quad \text{(S62)}$$

Eq. (S62) is equal to the second expression of Eq. (14) for $\nabla P(x,t)$.
